# Tackling *Pseudomonas aeruginosa* Virulence by Mulinane-Like Diterpenoids from *Azorella atacamensis*

**DOI:** 10.3390/biom10121626

**Published:** 2020-12-02

**Authors:** Onyedikachi Cecil Azuama, Sergio Ortiz, Luis Quirós-Guerrero, Emeline Bouffartigues, Damien Tortuel, Olivier Maillot, Marc Feuilloley, Pierre Cornelis, Olivier Lesouhaitier, Raphaël Grougnet, Sabrina Boutefnouchet, Jean-Luc Wolfender, Sylvie Chevalier, Ali Tahrioui

**Affiliations:** 1 Laboratoire de Microbiologie Signaux et Microenvironnement, Normandie Université, Université de Rouen Normandie, LMSM EA4312, 27000 Évreux, France; onyedikachi.azuama@etu.univ-rouen.fr (O.C.A.); emeline.bouffartigues@univ-rouen.fr (E.B.); damien.tortuel@etu.univ-rouen.fr (D.T.); olivier.maillot@univ-rouen.fr (O.M.); marc.feuilloley@univ-rouen.fr (M.F.); pcornel@vub.ac.be (P.C.); olivier.lesouhaitier@univ-rouen.fr (O.L.); sylvie.chevalier@univ-rouen.fr (S.C.); 2 Fédération de Recherche Sécurité Sanitaire, Bien-Être, Aliments Durables (SéSAD), Normandie Université, Université de Rouen Normandie, 27000 Évreux, France; 3 Department of Biological Sciences, Alex-Ekwueme Federal University, Ndufu Alike Ikwo PMB1010, Nigeria; 4 Équipe Produits Naturels, Analyses et Synthèses (PNAS), CiTCoM UMR 8038 CNRS, Faculté de Pharmacie, Université de Paris, 75006 Paris, France; sergio.ortiz.aguirre@gmail.com (S.O.); raphael.grougnet@parisdescartes.fr (R.G.); sabrina.boutefnouchet@parisdescartes.fr (S.B.); 5 Phytochemistry and Bioactive Natural Products, School of Pharmaceutical Science, University of Geneva, 1211 Geneva, Switzerland; Luis.Guerrero@unige.ch (L.Q.-G.); Jean-Luc.Wolfender@unige.ch (J.-L.W.); 6 Institute of Pharmaceutical Sciences of Western Switzerland (ISPSW), University of Geneva, CMU, 1211 Geneva, Switzerland

**Keywords:** mulinane-like diterpenoids, *Azorella atacamensis*, anti-virulence, *Pseudomonas aeruginosa*, membrane stiffness, ECFσ SigX

## Abstract

*Pseudomonas aeruginosa* is an important multidrug-resistant human pathogen by dint of its high intrinsic, acquired, and adaptive resistance mechanisms, causing great concern for immune-compromised individuals and public health. Additionally, *P. aeruginosa* resilience lies in the production of a myriad of virulence factors, which are known to be tightly regulated by the quorum sensing (QS) system. Anti-virulence therapy has been adopted as an innovative alternative approach to circumvent bacterial antibiotic resistance. Since plants are known repositories of natural phytochemicals, herein, we explored the anti-virulence potential of *Azorella atacamensis*, a medicinal plant from the Taira Atacama community (Calama, Chile), against *P. aeruginosa*. Interestingly, *A. atacamensis* extract (*Aa*E) conferred a significant protection for human lung cells and *Caenorhabditis elegans* nematodes towards *P. aeruginosa* pathogenicity. The production of key virulence factors was decreased upon *Aa*E exposure without affecting *P. aeruginosa* growth. In addition, *Aa*E was able to decrease QS-molecules production. Furthermore, metabolite profiling of *Aa*E and its derived fractions achieved by combination of a molecular network and in silico annotation allowed the putative identification of fourteen diterpenoids bearing a mulinane-like skeleton. Remarkably, this unique interesting group of diterpenoids seems to be responsible for the interference with virulence factors as well as on the perturbation of membrane homeostasis of *P. aeruginosa*. Hence, there was a significant increase in membrane stiffness, which appears to be modulated by the cell wall stress response ECFσ SigX, an extracytoplasmic function sigma factor involved in membrane homeostasis as well as *P. aeruginosa* virulence.

## 1. Introduction

*Pseudomonas aeruginosa*, a Gram-negative human opportunistic pathogen, is considered as a highly resistant bacterium and it has been enlisted with other multidrug resistant pathogens denoted with the acronym “ESKAPE” for *Enterococcus faecium, Staphylococcus aureus, Klebsiella pneumoniae, Acinetobacter baumannii, Pseudomonas aeruginosa*, and *Enterobacter* species [1,2]. *P. aeruginosa* is recognized as one of the major causes of hospital-acquired infections of great concern in cystic fibrosis, burns, indwelling catheters, and immunocompromised individuals [3,4]. *P. aeruginosa* possesses an intrinsic ability to adapt to various environmental conditions by dint of its high capability to establish biofilms, a sessile lifestyle representing a serious threat for individuals and public health [5,6,7]. Additionally, the pathogenicity of *P. aeruginosa* relies on its ability to produce a myriad of virulence factors, including pyocyanin, elastase B, alkaline phosphatase, rhamnolipids, siderophores, and toxins, among others [8,9]. The production of these virulence factors is controlled by a process called quorum sensing (QS), a cell density-dependent gene regulation mechanism, which involves the production and diffusion of signal molecules known as autoinducers [10]. *P. aeruginosa* possesses three interconnected Las, Rhl and Pqs QS circuits. The Las and Rhl circuits rely on *N*-(3-oxododecanoyl)-l-homoserine lactone (3-oxo-C_12_-HSL) and *N*-butanoyl-l-homoserine lactone (C_4_-HSL) autoinducers, respectively, with the Las system controlling the Rhl system. The Pqs system involves the production of molecules from the 4-hydroxy-2-alkylquinolines (HAQs) family, namely 3,4-dihydroxy-2-heptylquinoline (termed pseudomonas quinolone signal (PQS)). This third QS system in *P. aeruginosa* is also hierarchically dependent on the Las QS, which is at the top of the cascade [10,11].

In previous decades, antibiotics were successfully used in the treatment of several infectious diseases, but their increasing misuse has contributed to the emergence of multidrug resistant bacteria. Owing to the limitations of current antibiotics, there is an urgent need for the development of innovative alternative approaches to control and to circumvent accrued morbidity related to resistant bacterial infections [2,12]. The increased knowledge on bacterial molecular pathways involved in bacterial pathogenicity allowed the design of innovative alternative approaches that intend to interfere with virulence determinant production and/or their regulatory mechanisms [13,14,15]. For instance, the inhibition of QS circuitry, which is the master regulator involved in the expression of many virulence factors, represents a valuable adjuvant therapy that might be used to potentiate the efficacy of conventional antibiotics applied to treat early *P. aeruginosa* infections [14,15,16]. Moreover, the combination of anti-virulence therapy with already existing antibiotics can interfere with intrinsic antibiotic resistance, thus increasing their efficacy whilst extending their lifespan as well [16,17]. In the last decade, these approaches, known as anti-virulence therapy, have been increasingly explored and do not target bacterial growth or viability, hence bypassing the selective pressure that drives the emergence and development of antibiotic resistance [18,19].

In the framework of efforts to search for anti-virulence agents to tackle the pathogenicity traits and to interfere with intrinsic antibiotic resistance mechanisms of *P. aeruginosa*, a series of effective natural bioactive plant-derived molecules have been identified [20]. These natural phytochemicals include alkaloids, polyphenols, terpenoids, organosulphurs, phytosterols, and fatty acids among others [17,20,21]. Accruing evidence from the last two decades demonstrated that several natural bioactive products from plants or medicinal herbs possess therapeutic properties and are able to modulate microbial pathogenicity [20]. For instance, the Taira Atacama community (Calama, Chile) use infusions of the leaves and stems of *Azorella atacamensis* G. M. Plunkett and A.N. Nicolas (formerly known as *Mulinum crassifolium* Phil.) mainly for the treatment of diabetes, intestinal, bronchial and respiratory disorders [22,23,24,25,26]. This plant, popularly known as “*Chuquikandia*”, “*Chukikanlla*” or “*Chuquican*”*,* is an endemic shrub from Chile of about 15 cm with Austral-Antarctic affinities growing at high altitudes above 4000 m [22,23]. The medicinal plants belonging to the genus *Azorella* and *Mulinum* has been demonstrated to produce an interesting group of phytochemicals known as mulinane- and azorellane-like diterpenoids. To date, 49 natural mulinane and azorellane compounds are known to be present in the *Azorella* and *Mulinum* genera, as well as *Laretia* spp. [27]. In particular, 11 of them with only a mulinane skeleton have been isolated from *A. atacamensis* [27,28,29]. In 1990, Loyola and colleagues reported the first mulinane-like diterpenoids (mulinic and isomulinic acids) from *A. atacamensis* [30]. In the literature, mulinane and azorellane diterpenoids have been reported to possess a diverse range of biological activities, including antimicrobial, antiprotozoal, antidiabetic, spermicidal, antiulcer, and anti-inflammatory, among others [27,31,32,33]. However, very little is known about the anti-virulence activities of this unique interesting group of diterpenoids.

Based on the folk medicinal use of the plant *A. atacamensis,* the current study aims to investigate its anti-virulence properties against the pathogenicity of *P. aeruginosa.* Thus, the effect of *A. atacamensis* extract (*Aa*E) was evaluated against *P. aeruginosa* virulence using *Caenorhabditis elegans* and human lung infection models. Next, virulence factor production hindrance was ascertained by means of the quantification of pyocyanin, elastase and rhamnolipids. We further assessed the anti-QS effect of *Aa*E via *N*-acyl homoserine lactones (AHLs) and HAQs QS-molecules detection assays using biosensor strains. Subsequently, *Aa*E and all its fractions were subjected to metabolite profiling by liquid chromatography coupled to tandem mass spectrometry (UPHLC-HRMS/MS) with molecular networking for chemical composition assessment based on dereplication. The integration of bioactivity and fractions chemical composition results highlighted specific bioactive diterpenoids. Lastly, to investigate a possible mechanism of action of *Aa*E and its bioactive fractions enriched in mulinane-like diterpenoids, we assessed their interaction with *P. aeruginosa* membrane bilayers through fluorescence anisotropy measurements. The present study reveals an interesting singular group of phytochemicals from *A. atacamensis* represented by mulinane-like diterpenoids, which appears to display anti-virulence properties against *P. aeruginosa*.

## 2. Materials and Methods

### 2.1. Collection of Plant Material

Aerial parts of *Azorella atacamensis* were obtained from the Taira Atacama community (Calama, Chile) in September 2016 (21°44′43.546′′ S, 68°38′0.451′′ W, 3254 m above sea level), respecting Chilean Biodiversity Strategy and Nagoya Protocol legislations. The plant material was identified by Pr. Alicia Marticorena, Universidad de Concepción, Chile. Vouchers of *A. atacamensis* were deposited in the CONC Herbario, Universidad de Concepción, Chile (185885) and in the Herbarium of the Laboratory of Pharmacognosy, Paris Descartes University, France (MKOSB092015T2A04).

### 2.2. Preparation of Azorella atacamensis Extract and Fractionation

Aerial parts of *A. atacamensis* were dried under air, then pulverized using an electric grinder pulverisette 19 (Fritsch GmbH, Mahlen und Messen, Germany). Approximately 20 g of plant powder was subjected to pressurized solvent extraction using a Speed Extractor E-914 (Büchi, Fawil Switzerland) set at two extraction cycles with 15 min hold on time at 100 bar maximum pressure and 50 °C temperature, with ethyl acetate as a solvent. The solvent was removed under reduced pressure with a rotavapor and the obtained extract was labelled *Aa*E (5.8% of extraction yield). Subsequently, *Aa*E was solubilized at 10 mg/mL in 100% DMSO and stored at −20 °C until biological evaluation. For fractionation assays, a sample of 1.5 g of *Aa*E was subjected to a silica gel chromatography with a gradient of cyclohexane/ethyl acetate/methanol mixture solvent (10:0:0–0:8:2). Fourteen fractions labelled *Aa*F-(1-14) were obtained. For biological evaluation and LC-MS analysis, a solution at 10 mg/mL in 100% DMSO and a solution at 5 mg/mL in methanol grade HPLC, respectively, were prepared for each fraction.

### 2.3. Bacterial Strains, Media and Growth Conditions

The *Pseudomonas aeruginosa* H103 [34] and Δ*sigX* [35] used in this study are all derivatives of *P. aeruginosa* wild-type PAO1. Planktonic cultures were grown aerobically for 24 h at 37 °C in Luria–Bertani (LB) broth on a rotary shaker (180 rpm) from an initial inoculum adjusted to an O.D at 580 nm of 0.08. *Chromobacterium violaceum* CV026 [36] and *P. aeruginosa* PAO1 Δ*pqsA* CTX-*lux::pqsA* [37] were used as reporter strains for quorum sensing molecules detection. *C. violaceum* CV026 was grown at 28 °C in LB medium with orbital shaking (180 rpm). The PAO1 Δ*pqsA* CTX-*lux::pqsA* was cultured at 37 °C in LB medium supplemented with 125 µg/mL of tetracycline on a rotary shaker (180 rpm). The antibiotics stock solutions used in this study were sterilized by filtration through 0.22 μm filters, aliquoted into daily-use volumes and kept at −20 °C. Each set of experiments was performed at least three times.

### 2.4. Bacterial Growth Monitoring

To assess the effect of the *Aa*E on *P. aeruginosa* H103 strain growth kinetics, bacterial cultures were grown in the absence or presence of *Aa*E. Then, the growth of *P. aeruginosa* was monitored at 37 °C over the course of 24 h using the Spark 20 M multimode Microplate Reader, equipped with an active temperature regulation system (Te-Cool^TM^, Tecan Group Ltd., Männedorf, Switzerland). Absorbance at 580 nm was recorded every 15 min. The bacterial growth curve for untreated cultures (control condition) and treated cultures was determined by plotting the values against time.

### 2.5. Pyocyanin, Elastase and Rhamnolipids Quantification Assay

To evaluate pyocyanin production, *Pseudomonas aeruginosa* H103 cells untreated (DMSO 1%, *v*/*v*) or treated with *Aa*E at 25, 50 and 100 µg/mL were grown in Luria–Bertani (LB) broth in a 96-well microtiter plate at 37 °C for 24 h with shaking (180 rpm). After incubation for 24 h, cell growth was determined by measuring the O.D at 580 nm. The pyocyanin quantification assay was carried out as described previously [13,38]. Briefly, one volume of chloroform was used to extract free-cell supernatant samples. Then, ½ volume of 0.5 M HCl was added to the chloroform layer (blue layer). The absorbance of the HCl layer (red-pink layer) was recorded at 520 nm using the Spark 20 M multimode microplate reader controlled by SparkControl^TM^ software Version 2.1 (Tecan Group Ltd., Männedorf, Switzerland) and the data were normalized for bacterial cell density (A_580 nm_). The LasB elastolytic activity of *P. aeruginosa* H103 untreated or *Aa*E-treated cultures was assessed using previously described protocol [39] with minor modifications. In brief, a 50 μL aliquot of untreated or treated *P. aeruginosa* H103 cell free-supernatant was added to 950 μL of Elastin congo red buffer (100 mM Tris-HCl, 1 mM CaCl_2_ at pH 7.5) in 10 mg of Elastin Congo red substrate (Sigma Aldrich, Saint-Louis, MO, USA). The mixture was allowed to incubate at 37 °C for 18 h on a rotary shaker (180 rpm). Next, the reaction was stopped by adding 100 μL of 0.12 M EDTA. Insoluble substrate was sedimented by centrifugation and supernatant absorbance was recorded at 495 nm. The production of rhamnolipids was quantified following a methylene-blue-based method [40]. Briefly, 500 µL of cell-free supernatants from *P. aeruginosa* H103 grown in the absence or presence of *Aa*E at 25, 50 and 100 µg/mL were acidified to 2.3 ± 0.2 pH using 1 M HCl solution. Rhamnolipids were extracted twice with equal volume of ethyl acetate. The organic layer was dried in a speed vac rotatory evaporator and residue were dissolved in 500 µL of chloroform mixed with 50 µL of methylene blue solution (1.4% *w/v* methylene blue in 95% ethanol adjusted to 8.6 ± 0.2 pH by addition of 15 µL of a 50 mM borax buffer). The organic phase was transferred to a new tube and mixed with ½ volume of 0.2 M HCl solution before absorbance quantification at 638 nm.

### 2.6. Quorum Sensing-Molecules Extraction and Detection

The extraction of QS molecules from cultures of *P. aeruginosa* H103 strain untreated or treated with *Aa*E at 25, 50 and 100 µg/mL and their detection were performed following the techniques described in previous studies [41]. To detect AHLs, an overnight culture of the AHL indicator strain *C. violaceum* CV026 was diluted 1:100 in 5 mL LB medium and poured onto LB agar. Once the plates were dried, paper disks of 5 mm in diameter were placed on an agar plate and the AHL samples were applied. The assay plates were incubated overnight at 28 °C and the appearance of violacein pigment around the filter was determined. HAQs QS molecules were detected using the biosensor strain *P. aeruginosa* PAO1 Δ*pqsA* CTX-*lux*::*pqsA* as previously described [13,37] using a combined spectrophotometer/luminometer microplate assay. Briefly, 5 µL of the crude HAQ extracts obtained from *P. aeruginosa* H103 untreated or *Aa*E-treated conditions were diluted in LB medium. Aliquots of 100 μL of this dilution were mixed with 100 µL of 1:50 dilution of the PAO1 Δ*pqsA* CTX-*lux::pqsA* biosensor. Furthermore, bioluminescence and O.D at 580 nm were monitored in specialized white sided and clear bottomed 96-well microtiter plates (Perkin Elmer, Waltham, MA, USA) every 15 min at 37 °C for 24 h using the Spark 20 M multimode Microplate Reader (Tecan Group Ltd., Männedorf, Switzerland) controlled by SparkControl^TM^ software Version 2.1. Both HHQ and PQS synthetic standards (Sigma-Aldrich, Saint-Louis, MO, USA) prepared at 5 μM in 1:100 dilution of the HAQ biosensor strain served as positive controls, whereas the same dilution of biosensor strain served as a negative control. The activity of the reporter strain was quantified as relative luminescence unit (R.L.U)/O.D_580 nm_ for each culture suspension.

### 2.7. Membrane Fluidity Assessment by Fluorescence Anisotropy

Membrane fluidity analysis of *P. aeruginosa* H103 wild-type and Δ*sigX* mutant untreated or treated cultures with 25, 50 and 100 µg/mL of *Aa*E or 100 µg/mL of *Aa*F was performed via fluorescence anisotropy [13]. Bacterial cells were centrifuged (6300 rpm for 10 min at 25 °C). Pellets were washed twice with 10 mM MgSO_4_ before resuspension in the same wash solution to reach an O.D value of 0.1 at 580 nm. Then, 1 μL of 4 mM 1,6-diphenyl-1,3,5-hexatriene (DPH) dissolved in tetrahydrofuran was added to 1 mL aliquot of the diluted suspension and incubated at 37 °C for 30 min in the dark to allow the incorporation of the probe into the lipid membranes. Subsequently, fluorescence polarization was measured by the Spark 20M multimode Microplate Reader, equipped with an active temperature regulation system (Te-Cool^TM^, Tecan Group Ltd., Männedorf, Switzerland) with wavelengths of emission and excitation set at 425 and 365 nm, respectively. Calculation of fluorescence anisotropy (FA) values was performed according to following formula [42]:

$$r=\frac{\left(I1-I2\right)}{\left(I1+2\mathrm{xG}I2\right)}$$

where (*I*1) corresponds to the emission fluorescence intensity measured alongside and (*I*2) corresponds to the emission fluorescence intensity measured perpendicularly to light excitation plan. G corresponds to the G factor. The relationship between anisotropy and membrane fluidity is an inverse one, where decreasing anisotropy values correspond to a more fluid membrane and vice versa.

### 2.8. Virulence Attenuation of P. aeruginosa H103 and A. atacamensis Cytotoxicity Evaluation Using A549 Pulmonary Cells

Human pulmonary cell line A549 was used for between 27 and 38 passages. The cells were grown in tissue culture flasks containing Dulbecco’s modified Eagle’s medium (DMEM, Lonza, BioWhittaker^®^ supplied with glutamine), supplemented with 10% heat-inactivated (30 min, 56 °C) fetal bovine serum (FBS), and a combination of penicillin and streptomycin antibiotics (100 units/mL each). Prior to infection, A549 cells were treated with trypsin and washed with 1X PBS before plating into 24-well plates. The cells were grown at 37 °C in the presence of 5% CO_2_ with constant changing of media until 80–100% confluency of monolayer cells was reached. Virulence attenuation of *Aa*E on *P. aeruginosa* H103 was assessed via the enzymatic approach by measuring the release of soluble lactate dehydrogenase (Pierce^TM^ LDH Cytotoxicity Assay Kit, Thermo Scientific^TM^, Göteborg, Sweden) from the plasma membrane of lysed A549 cells into the culture supernatants. Briefly, untreated or *Aa*E (100 µg/mL)-treated *P. aeruginosa* H103 overnight culture cells (~8 × 10^7^ CFU/mL) were inoculated into confluent A549 monolayer cells grown on a 24-well tissue culture plate and incubated for 20 h. The A549 monolayer cells treated with 1X lysis buffer served as a positive control with maximum LDH release (100% lysis) as recommended by the manufacturer. Serum free A549 culture medium served as background LDH release. In addition, the LDH assay was used to assess the cytotoxicity of *Aa*E at 25, 50 and 100 µg/mL towards A549 monolayer cells upon direct exposure at 1, 3, 6, and 24 h post-inoculation. LDH is a stable enzyme present in different cell types, its release serves as an indicator of cell death. Furthermore, the integrity of A549 monolayers cells were constantly observed using an inverted photonic microscope (×400). Data represent the average of two independent experiments of duplicate assay.

### 2.9. Caenorhabditis Elegans Fast Killing Assay

*P. aeruginosa* virulence attenuation was assessed using a *Caenorhabditis elegans* fast-kill infection assay adapted from Blier and colleagues [43]. Briefly, 25 µL of cultures of *P. aeruginosa* H103 strain untreated and *Aa*E-treated at 25, 50 and 100 µg/mL adjusted to an O.D at 580 nm value of 0.08 were seeded on a 24-well plate containing peptone-glucose-sorbitol (PGS) agar. The control wells were inoculated with 25 µL of *Escherichia coli* OP50. Subsequently, the plate was incubated at 37 °C for 24 h to achieve bacterial lawns and transferred to 23 °C for 4 h. *C. elegans* wild-type Bristol strain N2 worms were maintained in nematode growth medium (NGM) agar plates and fed with *E. coli* OP50. For each assay, 15 to 20 L4-synchronized worms were added to the killing and control lawns and incubated at 23 °C. The survival of worms was recorded at 0, 4, 6, 8, and 24 h post-inoculation using an inverted Axiovert S100 optical microscope (Zeiss, Oberkochen, Germany).

### 2.10. HRMS Analysis

Chromatographic separation was performed on a Waters Acquity UPLC system hyphenated to a Q-Exactive Focus mass spectrometer (Thermo Scientific^TM^, Bremen, Germany), using a heated electrospray ionization (HESI-II) source and a CAD detector (Thermo Scientific^TM^, Bremen, Germany). The instrument was controlled using Thermo Scientific Xcalibur 3.1 software. The LC separation was done on a Waters BEH C18 50 × 2.1 mm, 1.7 μm column, using a linear gradient of 5−100% B over 7 min and an isocratic step at 100% B for 1 min. The mobile phase was (A) water with 0.1% formic acid or (B) acetonitrile with 0.1% formic acid. All analyses with flow rate, 600 μL/min and an injection volume of 2 μL. ESI parameters were as follows: source voltage, 3.5 kV (pos); sheath gas flow rate (N2), 55 units; auxiliary gas flow rate, 15 units; spare gas flow rate, 3.0; capillary temperature, 350.00 °C, S-Lens RF Level, 45. The mass analyzer was calibrated using a mixture of caffeine, methionine–arginine–phenylalanine–alanine–acetate (MRFA), sodium dodecyl sulfate, sodium taurocholate, and Ultramark 1621 in an acetonitrile/methanol/water solution containing 1% formic acid by direct injection. The data-dependent MS/MS events were performed on the three most intense ions detected in full scan MS (Top3 experiment). The MS/MS isolation window width was 1 Da, and the stepped normalized collision energy (NCE) was set to 15, 30 and 45 units. In data-dependent MS/MS experiments, full scans were acquired at a resolution of 35,000 FWHM (at *m*/*z* 200) and MS/MS scans at 17,500 FWHM both with an automatically determined maximum injection time. After being acquired in a MS/MS scan, parent ions were placed in a dynamic exclusion list for 2.0 s.

### 2.11. MS Data Treatment

All HRMS run data were converted from RAW (Thermo) standard data format to mzXML format using the MS Convert software, part of the ProteoWizard package [44]. The converted files were treated using the MZmine software suite version 2.53 [45]. For mass detection at MS^1^ level, the noise level was set to 1.0 × 10^4^ for positive mode and 1.0 × 10^5^ or negative mode. For MS^2^ detection, the noise level was set to 0 for both ionization modes. The ADAP chromatogram builder was used and set to a minimum group size of scans of 5, a minimum group intensity threshold of 1.0E6 (1.0 × 10^5^ negative), a minimum highest intensity of 1.0 × 10^6^ (1.0 × 10^5^ negative) and *m*/*z* tolerance of 8.0 ppm. The ADAP algorithm (wavelets) was used for chromatogram deconvolution. The intensity window S/N was used as an S/N estimator with a signal to noise ratio set at 25, a minimum feature height at 1.0 × 10^6^ (1.0 × 10^5^ negative), a coefficient area threshold at 100, a peak duration ranging from 0.02 to 1.0 min and the RT wavelet range from 0.02 to 0.08 min. Isotopes were detected using the isotope peak grouper with a *m*/*z* tolerance of 5.0 ppm, a RT tolerance of 0.02 min (absolute), the maximum charge set at 1, and the representative isotope used was the most intense. The peak list was gap-filled with the same RT and *m*/*z* range gap filler (*m*/*z* tolerance at 8 ppm). Eventually the resulting aligned peak list was filtered using the peak-list rows filter option to remove all the duplicates and all the features without a MS^2^ spectrum associated.

### 2.12. Mass Spectral Organization (Molecular Networks) and Taxonomically Informed Metabolite Annotation

A molecular network was constructed from the mgf file exported from MZmine, using the online workflow (https://ccms-ucsd.github.io/GNPSDocumentation/) on the GNPS website (http://gnps.ucsd.edu) [46]. The precursor ion mass tolerance was set to 0.02 Da with a MS/MS fragment ion tolerance of 0.02 Da. A network was then created where edges were filtered to have a cosine score above 0.7 and more than 6 matched peaks. The spectra in the network were then searched against GNPS’ spectral libraries. The library spectra were filtered in the same manner as the input data. All matches kept between network spectra and library spectra were required to have a score above 0.6 and at least 3 matched peaks. The works are available in the following links, negative: https://gnps.ucsd.edu/ProteoSAFe/status.jsp?task=c0e684e7e3d7493d9d99501f75b18b14. Positive: https://gnps.ucsd.edu/ProteoSAFe/status.jsp?task=c8a10978dc7d4879b08c21bec7c93a1a. Combined network extracts and fractions in negative mode: https://gnps.ucsd.edu/ProteoSAFe/status.jsp?task=839e6bd755e44fa2aae90901b62c527b.

Data visualization was achieved using Cytoscape version 3.8.0 [47]. The output of the GNPS was used to annotate against the in silico ISDB-DNP [48] and then the script for taxonomically informed metabolite annotation [49] was used to re rank and clean the output based on the species.

### 2.13. Statistical Analyses

All experiments were performed independently at least three times. Means and standard error of the means were calculated and plotted. Statistical data analyses were carried out with GraphPad Prism 8 using (two samples) two-tailed *t*-test. *C. elegans* survival curves were prepared using GraphPad Prism 8 to perform a statistical log-rank (Mantel-Cox) test. Significance was considered at ^★★★^, *p* = 0.0001–0.001; ^★★^, *p* = 0.001–0.01; ^★^, *p* = 0.01–0.05; NS: (not significant), *p* > 0.05.

## 3. Results

### 3.1. Pseudomonas aeruginosa Virulence Attenuation by Azorella atacamensis Extract

**Figure 1:**
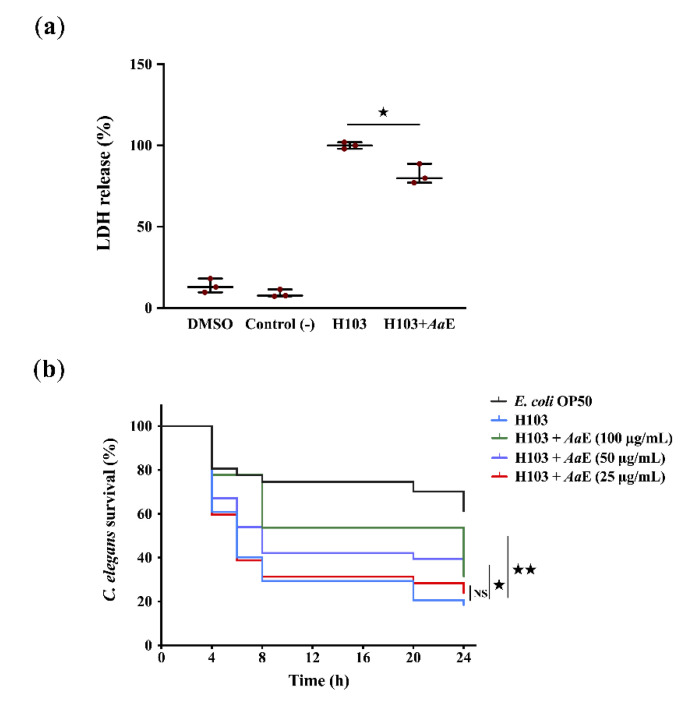
Anti-virulence effect of *A. atacamensis* extract (*Aa*E) against *P. aeruginosa* on A549 human lung cells and *C. elegans* infection models. (**a**) Virulence attenuation on *P. aeruginosa* by *Aa*E evaluated by using human A549 lung cells infection model. The presence of *Aa*E (100 µg/mL) significantly protected A549 lung cells from lysis after 20 h infection. Data are presented as the mean ± SEM values of three independent experiments performed in duplicate. ^★^*, p =* 0.01–0.05 (two-tailed *t-*test) versus untreated H103 cells. (**b**) *P. aeruginosa* H103 virulence attenuation in a *C. elegans* infection model by *Aa*E. Sixty L4-stage nematodes per experimental group were placed on lawns of *E. coli* OP50 (black) or H103 strain in the absence (red) or presence of *Aa*E at 25, 50 and 100 µg/mL (green). Alive *C. elegans* nematodes were scored at 4, 6, 8, 20, and 24 h after the start of the assay. ^★★^, *p =* 0.001–0.01; ^★^, *p =* 0.01–0.05; NS: (not significant), *p >* 0.05 (log-rank [Mantel-Cox] test) versus control condition.

*Azorella atacamensis* extract’s (*Aa*E) anti-virulence effect was explored using human A549 pulmonary cells and *Caenorhabditis elegans* infection models. In the human lung A549 cell model, lactate dehydrogenase (LDH) release by A549 cells into the culture medium was recorded after 20 h of infection with *Pseudomonas aeruginosa* H103, a prototrophic strain of the well-known wild-type PAO1 [34], untreated or treated previously with *Aa*E at 100 µg/mL. As a result, LDH release decreased significantly (18%; *p* = 0.01–0.05) when A549 cells were infected with H103 cultures exposed to *Aa*E at 100 µg/mL in comparison with the control condition (untreated H103 cultures) (Figure 1a). To confirm this virulence attenuation, in vivo fast killing of *C. elegans* in contact with *p. aeruginosa* H103 cultures untreated or treated previously with *Aa*E was performed. The results show a drastic decrease in the survival rate of *C. elegans* (18.5%; *p* = 0.001–0.01) when spread on a lawn of H103 strain 24 h post-incubation in comparison with nematodes’ survival fed with *Escherichia coli* OP50 (Figure 1b). Interestingly, we recorded a significant survival improvement (31.5%; *p* = 0.001–0.01) of the worms seeded in the presence of *P. aeruginosa* exposed to *Aa*E at 100 µg/mL as compared to the control condition (worms spread on a lawn of untreated H103 culture), suggesting that *Aa*E confers a protection against *P. aeruginosa* killing. Altogether, these data indicate that *Aa*E attenuates *P. aeruginosa* virulence.

### 3.2. Azorella atacamensis Extract Decreases Pyocyanin, Elastase, and Rhamnolipids Production in Pseudomonas aeruginosa

**Figure 2:**
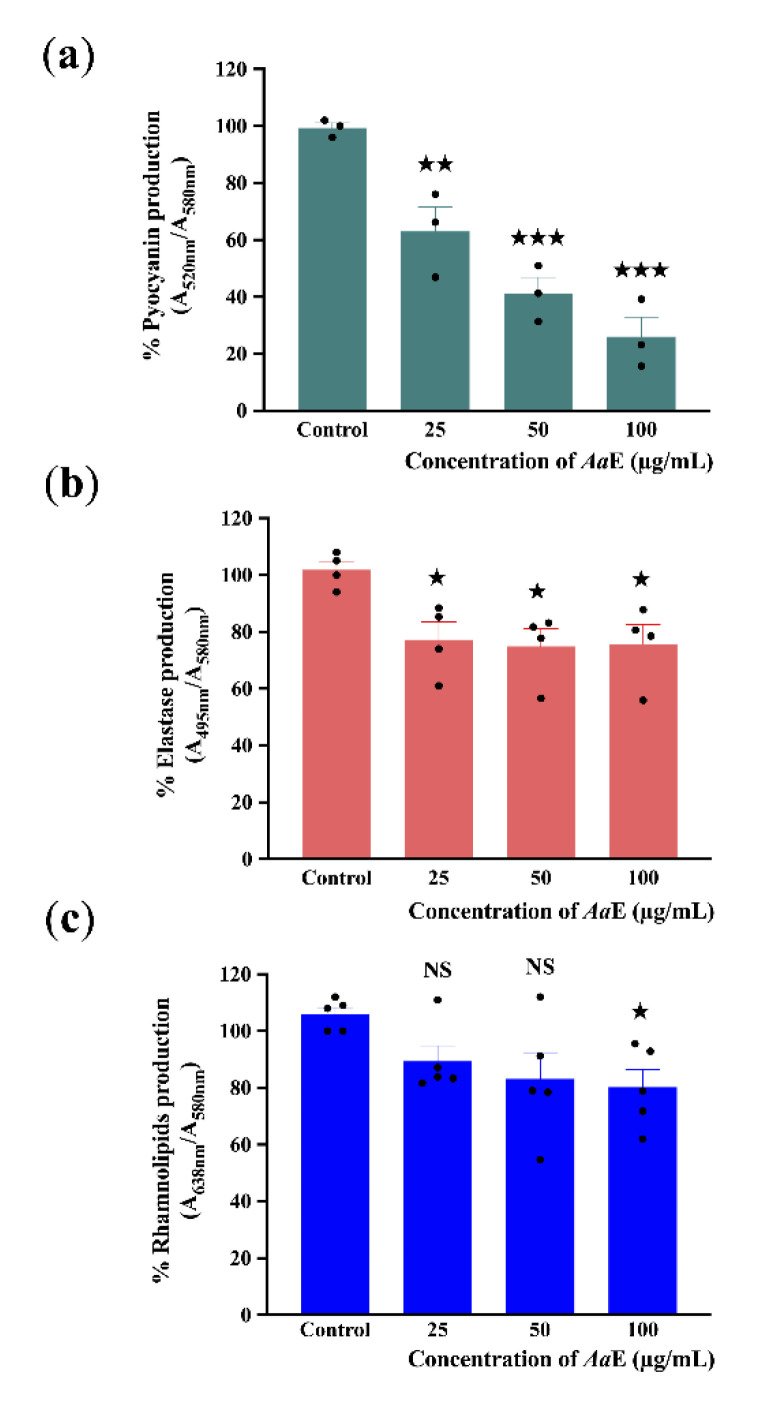
Effect of *Aa*E on *P. aeruginosa* pyocyanin (**a**), elastase (**b**), and rhamnolipid (**c**) virulence factor production. *P. aeruginosa* was grown in the absence or presence of *Aa*E at 25, 50, and 100 µg/mL. All results were normalized against cell density (A_580nm_). Data represent mean ± SEM of at least three independent biological assays. Significant differences between mean values versus control were assessed via GraphPad Prism 8 *t-*test calculator. ^★★★^, *p =* 0.0001–0.001; ^★★^, *p =* 0.001–0.01; ^★^, *p =* 0.01–0.05; NS: (not significant), *p* > 0.05.

As a result of *P. aeruginosa* virulence attenuation, we further evaluated the effect of *Aa*E on virulence factor production including pyocyanin, elastase, and rhamnolipids. Pyocyanin is a bluish-green secondary phenazine metabolite responsible for cellular damage and necrosis in acute infections owing to reactive oxygen intermediate production [50]. *P. aeruginosa* H103 strain was grown in the absence or presence of *Aa*E at 25, 50, and 100 µg/mL. Subsequently, quantification of pyocyanin production levels from the culture supernatants was performed. The results show that *Aa*E at 100 µg/mL inhibits pyocyanin production significantly (about 70%; *p* = 0.0001–0.001) as compared to the control condition (Figure 2a). Moreover, a concentration-dependent reduction in pyocyanin pigment production was observed. This result demonstrates that *Aa*E can function as a strong inhibitor of pyocyanin production in *P. aeruginosa*. Additionally, the impact of *Aa*E on elastase and rhamnolipid production by *P. aeruginosa* was also examined. Elastase and rhamnolipids are important virulence factors for tissue invasion and infection. The elastolytic activity was reduced (by about 24%; *p* = 0.01–0.05) when H103 strain was exposed to 25, 50, and 100 µg/mL concentrations of *Aa*E (Figure 2b), whereas rhamnolipids production in H103 was only significantly decreased to a level of about 20% (*p* = 0.01–0.05) at the highest *Aa*E concentration tested in comparison with untreated control condition (Figure 2c). These data indicate that *Aa*E contains bioactive compounds that thwart the production of virulence factors that are known to be controlled by the QS regulatory system in *P. aeruginosa*.

### 3.3. Azorella atacamensis Extract Modulates QS-Molecules Production

**Figure 3:**
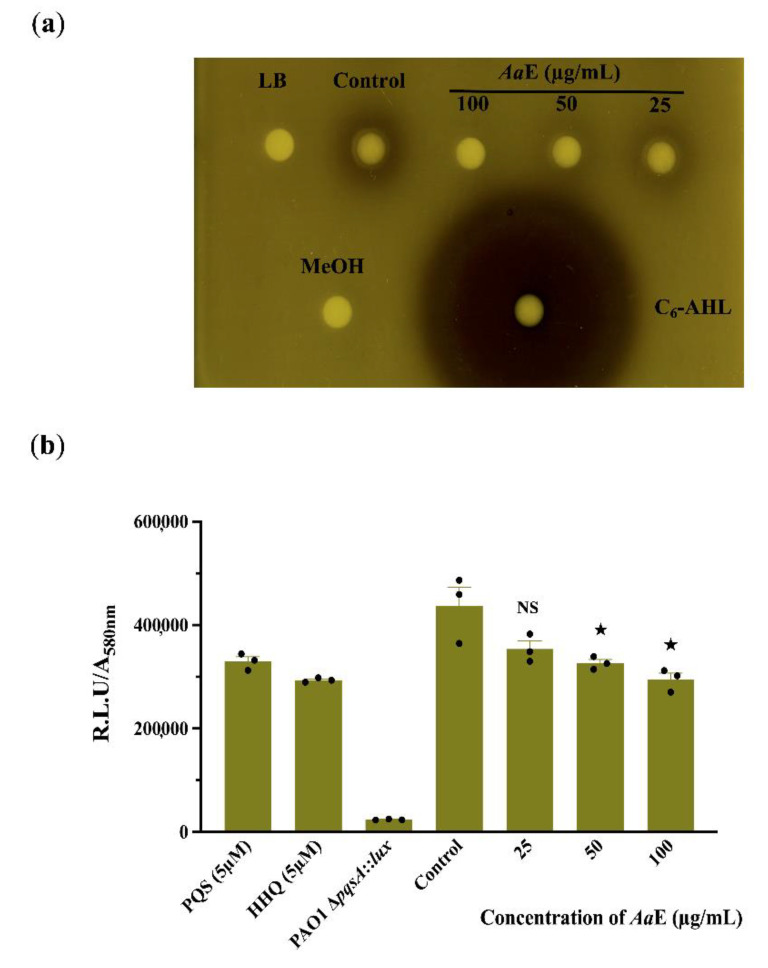
Effect of *Aa*E on QS-molecule production. (**a**) Crude *N*-acyl homoserine lactones (AHLs) extracted from H103 cultures untreated (control) or treated with *Aa*E at 25, 50 and 100 µg/mL were visualized using *C. violaceum* CV026 biosensor agar plate assay. MeOH, methanol (negative control). LB, AHLs crude ethyl acetate extract from LB medium (negative control). C_6_-HSL, synthetic AHL (positive control). (**b**) Normalized mean maximal bioluminescence output from the 4-hydroxy-2-alkylquinoline (HAQ)-biosensor strain in the presence of crude ethyl acetate HAQs extracts prepared from cultures of H103 treated with different concentrations of *Aa*E compared to positive control conditions (HHQ and PQS at 5 µM) and to negative control conditions (HAQs crude ethyl acetate extract from HAQ-biosenor strain). Data represent mean ± SEM of at least three independent biological assays. Significant differences between mean values versus control were assessed via GraphPad Prism 8 *t-*test calculator. ^★^, *p =* 0.01–0.05; NS: (not significant), *p >* 0.05.

Since QS systems regulate virulence determinant production in *P. aeruginosa*, we assumed that the *Aa*E interfere with the *N*-acyl homoserine lactones (AHLs) and/or hydroxy-alkyl quinolines (HAQs) QS-molecules production. The AHLs extracts from *P. aeruginosa* H103 treated with *Aa*E at 50 and 100 µg/mL activated the *Chromobacterium violaceum* CV026 indicator organism to a lesser extent than did the crude AHLs extracted from untreated H103 cultures (Figure 3a). This result reveals that H103 strain produced lower levels of AHLs in the presence of *Aa*E, suggesting that it may possess bioactive compounds interfering with the QS regulatory system. In addition, the production of HAQs QS-molecules was assessed via bioluminescence measurements using PAO1 ∆*pqsA* CTX-*pqsA*::*lux* bio-reporter strain. The results show a dose-dependent decrease in HAQs molecule production; however, this production interference was significant only at 50 and 100 µg/mL of *Aa*E as compared to the control condition (Figure 3b). Taken together, these findings imply that the attenuation of virulence factor production by the *Aa*E occurs at least through the interference of QS-molecule production.

### 3.4. Azorella atacamensis Extract Did Not Affect Pseudomonas aeruginosa Growth and Did Not Exert Cytotoxic Effect on A549 Lung Cells

**Figure 4:**
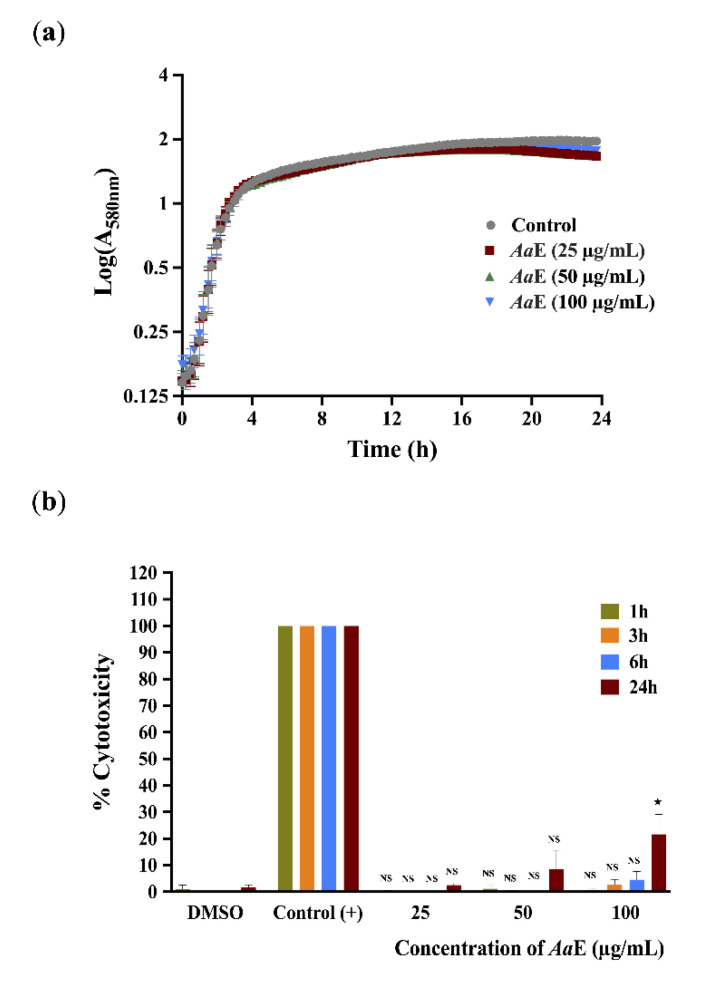
(**a**) Effect of *Aa*E on *P. aeruginosa* H103 growth. *P. aeruginosa* H103 was grown in the absence or presence of *Aa*E at 25, 50 and 100 µg/mL. Data represent the mean ± SEM of three independent biological assays. (**b**) Cytotoxic effect of *Aa*E on A549 human lung line cells. *Aa*E was assayed at 25, 50 and 100 µg/mL. LDH release was determined at 1, 3, 6, and 24 h. DMSO was used as a vehicle control. The control (+) corresponds to A549 monolayer cells treated with 1X lysis buffer, which served as a positive control with maximum LDH release (100% lysis). Data are displayed as the mean ± SEM values of three independent experiments performed each in duplicate. Statistics were achieved by a two-tailed *t* test: ^★^, *p =* 0.01–0.05; NS: (not significant), *p >* 0.05.

To further exonerate alterations in bacterial growth as the cause of the observed anti-virulence properties, growth kinetics of H103 strain untreated or treated with *Aa*E at 25, 50, and 100 µg/mL concentrations were monitored by recording the O.D at 580 nm every 15 min during 24 h time period. The obtained results show that the *Aa*E had no effect on *P. aeruginosa* growth, suggesting no antibacterial activity, at least at the assayed concentrations (Figure 4a), thus the anti-virulence properties of *Aa*E were achieved without disturbing cell growth. In addition, we ascertained the cytotoxic effect of *Aa*E at 25, 50 and 100 µg/mL over a period of 1, 3, 6 and 24 h using the A549 human pulmonary cell line. The A549 monolayer cells treated with 1X lysis buffer served as a positive control to determine the maximum LDH release (100% lysis). As shown in Figure 4b, no significant cytotoxicity was observed up to 6 h post-incubation when compared with untreated condition (DMSO was used as a vehicle control). However, a moderate, but significant, cytotoxic effect (24.5% of A549 cell lysis; *p* < 0.05) was observed when *Aa*E was tested at the highest concentration (100 µg/mL) and after 24 h exposure.

### 3.5. Azorella atacamensis Extract Fractionation and Dereplication

The *Aa*E was subjected to fractionation using a silica gel column, and fourteen fractions labelled *Aa*F-(1–14) were obtained. To assess the chemical composition of the extract and fractions, metabolite profiling was performed using an UHPLC-PDA-CAD-ESI-HRMS/MS in negative (NI) and positive ionization modes. These analyses provided high-resolution accurate molecular ion features (MS^1^) and fragmentation patterns (MS^2^) for the detected ions. The charged aerosol detector (CAD) trace provided semi quantitative information of the compounds ratio in the samples [51]. The dereplication process was performed based on the ESI negative analysis, on account of the good profile similarity with the CAD trace, and the ratio of the major compounds observed (Supplementary Figure S1). The spectrometric data were treated in MZmine software [45] and uploaded to the GNPS platform [46] for construction of the molecular networks (MN, based on MS^2^). Then, they were subjected to dereplication based on comparison with in silico fragmentation spectra of a large natural products structural database [48]. The results were refined by a taxonomical informed metabolite annotation [49] restricted to the species *Azorella atacamensis*, allowing us to improve the accuracy of the putative identification.

**Figure 5:**
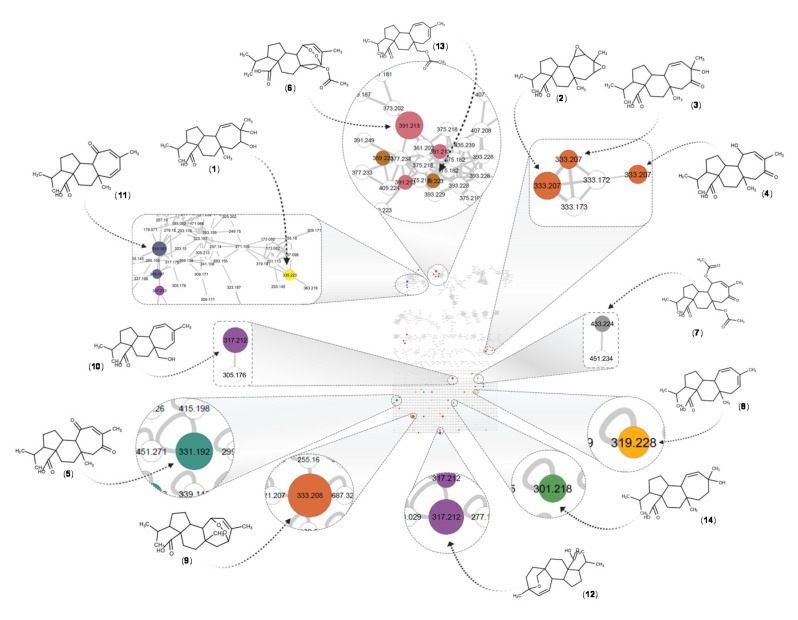
Molecular network in negative ionization mode for the *Aa*E. The displayed structures correspond to the fourteen putatively identified compounds (1–14, see Table 1). The identification of mulinic acid (9) was confirmed by isolation. Numbers inside the nodes correspond to the precursor mass for each feature, and the size is proportional to the intensity of each ion in the total ion current chromatogram of the extract. Colors represent the molecular formulas for all the reported compounds in the genus *Azorella* and *Mulinum*. The full MN and molecular formula color codes are shown in Supplementary Figure S2.

According to the Dictionary of Natural Products (DNP v29.1), a total of thirty-seven compounds belonging to mulinane-type and azorellane-type diterpenoids have been reported for the genera *Mulinum* and *Azorella* (Supplementary Table S1). Eight of these compounds have been isolated from the species *Mulinum crassifolium*, the former name of *Azorella atacamensis* [52]. This chemical family of compounds presents several isomers for a single molecular formula and exact mass. For example, for a [M-H]^−^ at *m*/*z* 333.2076 with a molecular formula C_20_H_30_O_4_, four different annotations are possible based on the previously reported compounds. Additionally, in the chromatogram, four different peaks were detected with the same accurate mass but different retention times. To identify possible isomers of the previously reported compounds (Supplementary Table S1), an additional dereplication step consisting in the extraction of all detected formula at the MS^1^ level was performed, based on accurate mass and heuristic filtering [53]. A total of fifty-eight features were putatively identified using the custom database search algorithm in the MZmine software. These results were mapped on the MN-NI, colour coded according to the molecular formula, allowing for directly spotting all the ions that possibly belonging to mulinane-type and azorellane-type diterpenoids (Figure 5, Supplementary Figure S2). This additional layer of information shows that besides the compounds annotated based on the in silico match of each individual MS^2^ spectra, there were still several other features that could correspond to similar compounds.

**Figure 6:**
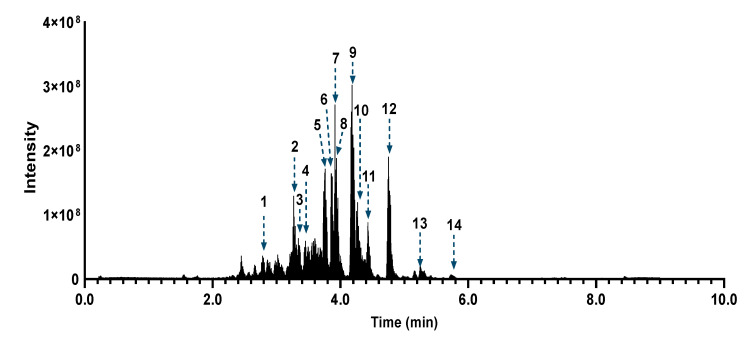
Total ion current chromatographic trace (ESI negative) showing the 14 putatively identified compounds in the ethyl acetate extract of *Azorella atacamensis* (*Aa*E).

After such dereplication of the MN, fourteen compounds (1–14), all diterpenes derivatives, were putatively identified (Table 1, Figure 6). Compounds (2), (6), (7), (8), (9), (12) and (13) have been previously reported in *A. atacamensis* species. Mulinic acid (9) was unambiguously identified by isolation and spectral analysis (Supplementary Figure S3). To the best of our knowledge, the diterpenes (1), (3), (4), (5), (10), (11), and (14) are reported for first time in the *A. atacamensis* species; however, they have been previously isolated from plants of the genus *Azorella* and *Mulinum* (Table 1). This reinforces the confidence of the annotation process and the putative identification through the concordance of the chemistry and taxonomy [54,55]. Based on the CAD trace (Supplementary Figure S1), the main compounds present in the *Aa*E are 11,14-dioxo-12-mulinen-20-oic acid (5), 17-acetoxymulinic acid (6), 13-hydroxy-11-mulinen-20-oic acid (8), mulinic acid (9) and mulinenic acid (12).

### 3.6. Bioactivity Assesement and Correlation with the Fractions Chemical Composition

**Figure 7:**
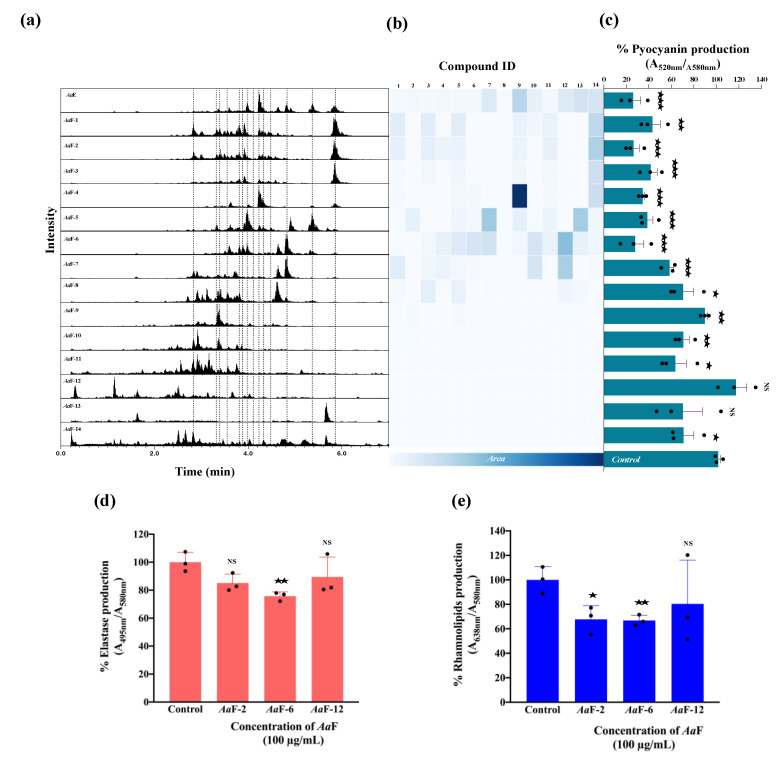
(**a**) Total ion current chromatograms of *Aa*E and its fractions (*Aa*F). Dashed lines indicate the retention time of the dereplicated compounds (1 to 14, left to right). (**b**) Heatmap based on the ESI-negative total ion current chromatogram peak integration of dereplicated compounds in the *Aa*E and all *Aa*F. (**c**) Effect of *Aa*E and each of the fourteen *Aa*F on the production of pyocyanin by *P. aeruginosa*. (**d**) Effect of *Aa*F-2 and *Aa*F-6 (diterpene-enriched fractions) and *Aa*F-12 on *P. aeruginosa* elastase and (**e**) rhamnolipids production. Data represent mean ± SEM of at least three independent biological assays. Significant differences between mean values versus control conditions were assessed via online graph pad *t-*test calculator. ^★★★^, *p =* 0.0001–0.001; ^★★^, *p =* 0.001–0.01; ^★^, *p =* 0.01–0.05; NS: (not significant), *p >* 0.05.

To correlate the bioactivity to the putatively identified chemical compounds and the general composition of *Aa*E and its derived fractions (*Aa*F), the chromatographic information and the MN in NI mode (Supplementary Figure S2) were further explored. Figure 7a shows the total ion current chromatographic traces in NI mode for the extract and all fractions. The peak areas corresponding to the dereplicated compounds (1–14) were used to generate the heatmap shown in Figure 7b. The production of pyocyanin by *P. aeruginosa* H103 upon exposure to *Aa*E and all its *Aa*F is shown in Figure 7c. The interpretation of the heatmap (Figure 7b) together with the bioactivity results (Figure 7c) revealed a possible correlation between the composition and the bioactivity of the samples. Based on the bioactivity results, fractions *Aa*F-1 to *Aa*F-6 were considered as the most active, whereas fractions *Aa*F-7 to *Aa*F-14 were considered less active. No clear pattern was recognized in the distribution of compounds (1–14) across the fractions but a variation in their concentration crosswise was observed. In general, it appears that there is a correlation between the pyocyanin inhibition and the concentration of the diterpenoids (1–14), hence, as their concentration decreases, the production of pyocyanin increases. Fractions *Aa*F-2 and *Aa*F-6 were shown to be the most active fractions. Interestingly, a difference in their composition was observed. A careful inspection of the adjacent fractions, *Aa*F-1 and *Aa*F-3, showed that their overall composition is practically similar to that of *Aa*F-2, but the concentration of the compounds varies, especially for the 11,13-mulinadien-20-oic acid (14). This diterpene is probably responsible for the observed anti-pyocyanin activity. The diterpene profile of *Aa*F-5 and *Aa*F-7 fractions varies considerably from that of *Aa*F-6. For this case, it seems that compounds 11,12-epoxy-13-mulinen-20-oic acid (10) and mulinenic acid (12), are not directly linked to the observed anti-pyocyanin activity, because both compounds are almost present in the same concentration in *Aa*F-7, which displays less important anti-pyocyanin activity. For *Aa*F-6, the overall complex mixture of diterpenes seems to be responsible for the biological activity and no specific diterpene could be highlighted based in this differential composition assessment of the concomitant fractions. In addition, the production of elastase and rhamnolipids was significantly decreased in the presence of *Aa*F-6 as compared to the control condition (Figure 7d,e). The same tendency of results was observed for *Aa*F-2, although the decrease in elastase production was not statistically significant. However, the fraction *Aa*F-12 did not affect elastase activity and rhamnolipid content in *P. aeruginosa* H103 (Figure 7d,e).

The chemical composition of fractions *Aa*F-9 to *Aa*F-14 was examined according to the combined MN of *Aa*E and *Aa*F (Supplementary Figure S4). These fractions were clearly different from *Aa*F-1 to *Aa*F-6, since no diterpenes were detected. Some clusters highlighted polar constituents, mainly present in fractions from *Aa*F-11 to *Aa*F-14. Additionally, many low-intensity ions in a large retention time range were observed within these fractions. These ions, barely seen in the chromatogram of *Aa*E (Figure 6 and Figure 7), correspond to minor compounds, which was consistent with the CAD trace (Supplementary Figure S1). The in silico annotation process suggested that these ions are small fatty acyl saccharides, polyphenols and small terpene derivatives. Altogether, these data suggest that the mulinane-type diterpenoids might possibly be responsible for the observed anti-virulence activity against *P. aeruginosa*.

### 3.7. Azorella atacamensis Derived Compounds Disturb Pseudomonas aeruginosa Cell Envelope Homeostasis

**Figure 8:**
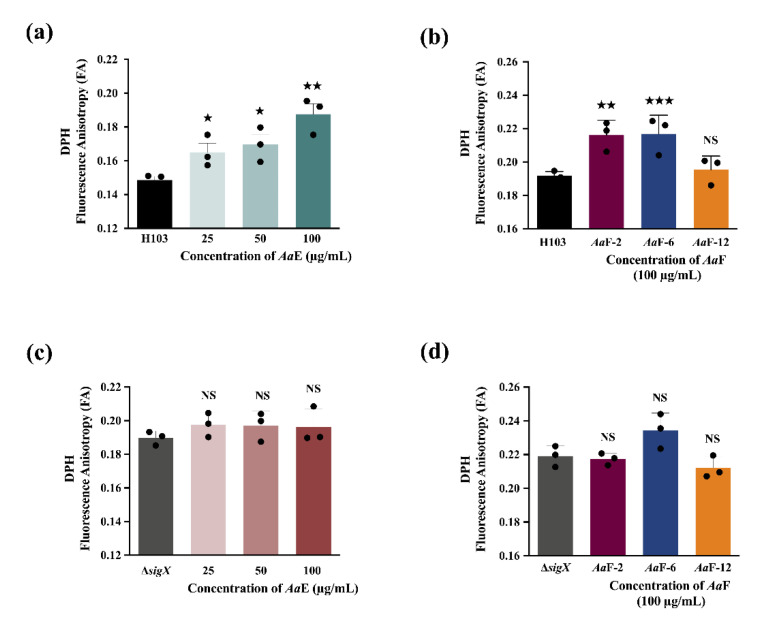
(**a**) Fluorescence anisotropy (membrane fluidity) measurements in *P. aeruginosa* H103 wild-type exposed to various concentrations of *Aa*E, and (**b**) *Aa*F-2 and *Aa*F-6 (diterpene-enriched fractions) and *Aa*F-12 at 100 µg/mL compared to H103 untreated condition. (**c**) Fluorescence anisotropy measurements in *P. aeruginosa* Δ*sigX* mutant exposed to various concentrations of *Aa*E, and (**d**) *Aa*F-2 and *Aa*F-6 (diterpene-enriched fractions) and *Aa*F-12 at 100 µg/mL compared to the Δ*sigX* untreated condition. Significant differences between mean values versus control were assessed via *t-*test using GraphPad Prism 8. ^★★★^, *p =* 0.0001–0.001; ^★★^, *p =* 0.001–0.01; ^★^, *p =* 0.01–0.05; NS: (not significant), *p >* 0.05.

The bacterial cell envelope is the first protective barrier against environmental assaults, hence we sought to ascertain the effect of *A. atacamensis* extract (*Aa*E) and representative of its fractions (*Aa*F) on *P. aeruginosa* envelope homeostasis. *P. aeruginosa* H103 cells untreated or treated with *Aa*E at 25, 50, and 100 µg/mL concentrations were investigated for membrane fluidity variation by measuring fluorescence anisotropy (FA) using 1,6-diphenyl-1,3,5-hexatriene (DPH) fluorescent probe. Interestingly, the results show increased FA values in a dose-dependent manner (Figure 8a). The FA value recorded at 100 µg/mL of *Aa*E was highly significant (0.188 ± 0.006; *p* = 0.001–0.01) as compared with the FA value of the untreated control condition (0.149 ± 0.002), revealing a significant decrease in membrane fluidity (approximately 26.2% membrane rigidification). In addition, membrane fluidity measurements were assessed in *P. aeruginosa* wild-type H103 strain upon exposure to *Aa*F-2, *Aa*F-6 (diterpene-enriched fractions) and *Aa*F-12 at 100 µg/mL (Figure 8b). Interestingly, while the FA value obtained upon exposure of H103 cells to *Aa*F-12 was unaffected as compared to the untreated control condition, the fractions *Aa*F-2 and *Aa*F-6 induced a significant increase in FA values (increased membrane stiffness). This alteration or damage of the *P. aeruginosa* cell envelope upon exposure to *Aa*E or *Aa*F may possibly lead to physiological changes and thus a cell envelope stress response. In *P. aeruginosa*, the extracytoplasmic function sigma factor (ECFσ) SigX is known to be involved in cell envelope stress response [57]. Further experiments were conducted to validate the involvement of the ECFσ SigX on membrane fluidity modulation in the presence of the *Aa*E or *Aa*F. As shown in Figure 8c, Δ*sigX* mutant of *P. aeruginosa* exposed to *Aa*E at 25, 50 and 100 µg/mL showed no differences in FA values. Moreover, a similar result was observed when Δ*sigX* mutant cells were exposed to *Aa*F-2, *Aa*F-6 and *Aa*F-12 fractions (Figure 8d). All together, these data suggest that *Aa*E, *Aa*F-2 and *Aa*F-6 (diterpene-enriched fractions) disturb *P. aeruginosa* membrane homeostasis. It appears that the bioactive compounds belonging to mulinane-like diterpenoids putatively identified in *Aa*E, *Aa*F-2 and *Aa*F-6 can interact with lipid membranes, triggering increased stiffness, and these compounds might modulate directly or indirectly through the ECFσ SigX an impact on *P. aeruginosa* H103 virulence.

## 4. Discussion

The anti-virulence therapy approaches pursue interventions to find new means that do not possess bacteriostatic or bactericidal effects [14,15,18,19], to minimize the emergence or development of bacterial resistance and undesirable side effects on the resident microbiota. Plants are widely known as repositories of medicinal bioactive phytochemicals including alkaloids, polyphenols, terpenoids, organosulphurs, phytosterols, lipids, and other compounds against bacterial infections [20,21]. For centuries, the leaves, stems, roots and bark of plants as potions or infusions have traditionally been used in the treatment of various ailments prior to the development of pharmacy. For instance, the traditional medicinal uses reported for the plant *Azorella atacamensis* G.M. Plunkett and A.N. Nicolas (synonym of *Mulinum crassifolium*) [52], belonging to the *Apiaceae* family, include for the common cold, coughing, pain, relaxing and diabetes, mainly to treat respiratory, gastro-intestinal and urinary disorders and inflammation [22,23,25]. Therefore, a range of pharmacological activities were reported for bioactive compounds isolated from the genus *Azorella*, such as antimicrobial, antiprotozoal, gastroprotective, and anti-inflammatory, among others [27,28]. However, in reviewing the literature, no data were found on the anti-virulence activity of *A. atacamensis*, a medicinal plant used by the people of the Taira Atacama community (Calama, Chile), against the human opportunistic pathogen *Pseudomonas aeruginosa*.

The current study found that *A. atacamensis* extract (*Aa*E) attenuates the pathogenicity of *P. aeruginosa* towards A549 human lung cells and *Caenorhabditis elegans* nematode infection models. Accordingly, the results also demonstrate significant reduction in the production of several virulence factors, such as pyocyanin, elastase and rhamnolipids, which are known to degrade host tissues and evade immune response activities, further exacerbating the spread of the infection [8,9]. For instance, the virulence factor pyocyanin is a bluish-green phenazine compound that generates reactive oxygen species such as hydrogen peroxide, which results in the deterioration and decrease in function of the lungs of cystic fibrosis patients leading to increased mortality [50,58,59,60]. Importantly, *Aa*E drastically hindered pyocyanin production (by more than 70% when added at 100 µg/mL), which may have consequences regarding the cytotoxic effects and the full virulence of *P. aeruginosa* during infections related to airways in cystic fibrosis. It is worth mentioning, however, that the effect of *Aa*E on elastase and rhamnolipids production is rather moderate. In *P. aeruginosa*, the expression of these virulence factors is under the control of the QS regulation system [10]. The inhibition of QS is recognized as an alternative therapeutic strategy for treatment of *P. aeruginosa* [12,14,15]. Accordingly, our data showed that *Aa*E interferes with *N*-acyl-homoserine lactones (AHLs) and 4-hydroxy-2-alkylquinolines (HAQs) QS-molecules production. The two biosensor strains, *C. violaceum* CV026 and *P. aeruginosa* PAO1 Δ*pqsA* CTX-*lux*::*pqsA*, revealed that *Aa*E reduces the production of the QS molecules AHLs and HAQs, respectively. In this regard, we can assume that the observed virulence attenuation displayed by *Aa*E may be partly because of the interference with the interconnected QS system cascade. A significant diversity of natural plant-derived QS inhibitors, including phenolic acids, flavonoids, terpenoids, coumarins, tannins, and sulfur-containing compounds have been reported in the literature [20,21]. Numerous studies documented in silico, in vitro, and in vivo evidence about the mode of action of these AHL- and HAQ-mediated QS inhibitors [15,21]. The understanding of phytochemicals mechanisms of action can lead to the development of innovative cocktail compositions of plant-derived molecules exhibiting different modes of action. As mentioned above, it is believed that targeting bacterial pathogenicity rather than the basic life processes yields a subtle approach to infectious disease control [16,17,19]. The data obtained in this study showed that *Aa*E, when added at 100 µg/mL, did not affect the growth of *P. aeruginosa*, which can minimize the selective pressure that enhances resistance development, as is the case for conventional antibiotics. However, further experiments will prove whether repeated exposure to *Aa*E can induce *P. aeruginosa* resistance. It should be noted that a number of natural and semisynthetic compounds derived from *Azorella*, *Laretia*, and *Mulinum* genera exhibited antimycobacterial and antimicrobial activities [27,33,61,62]. In addition, *Aa*E exhibited minimal cytotoxicity towards A549 lung cells line, which is a good attribute that could be promising for its therapeutic application and clinical relevance. Collectively, our findings indicate that the medicinal plant *A. atacamensis* appears to produce bioactive metabolites that can thwart *P. aeruginosa* virulence without disturbing its growth.

To get further insights on the bioactive phytochemicals responsible for the observed anti-virulence activity against *P. aeruginosa*, a liquid chromatography coupled to tandem mass spectrometry (UPHLC-HRMS/MS) analysis combined with molecular networking data processing was applied to *Aa*E and its derived fractions [63,64]. Interestingly, fourteen compounds were putatively identified in *A. atacamensis*. These phytochemicals belong to a well-known class of plant secondary metabolites called terpenoids. Terpenoids, also known as isoprenoids, are structurally diverse and their fundamental building block relies on the isoprene unit (C_5_H_8_). For instance, monoterpenoids are based on two isoprene units (C_10_H_16_) and the other terpenoids have multiples of C_5_ units (C_15_, C_20_, C_25_, C_30_, etc.) [65]. The terpenoids present in *A. atacamensis* extract and its derived fractions analyzed herein were categorized as diterpenoids. Especially, the fourteen identified compounds displayed mulinanes-like skeletons which are known to be produced by species of the genera *Mulinum*, *Azorella*, and *Laretia* [27,29,31,54,55,66]. To date, thirty-seven mulinanes-like diterpenoids have been reported from natural sources. It is noteworthy that eleven of them were isolated from *A. atacamensis* [27]. While seven of the dereplicated compounds in the *Aa*E and fractions were previously isolated from *A. atacamensis*, the other seven mulinanes were isolated from *Azorella compacta*, *Azorella madreporica*, *Azorella trifurcata*, and *Mulinum spinosum* species. Hence, based on the chromatographic traces and dereplication processes conducted in this study, the mulinane-like diterpenoids 11,14-dioxo-12-mulinen-20-oic acid, 17-acetoxymulinic acid, 13-hydroxy-11-mulinen-20-oic acid, mulinic acid and mulinenic acid are most likely the major compounds present in the ethyl acetate extract of *A. atacamensis*. Remarkably, the 11,14-dioxo-12-mulinen-20-oic acid compound was previously isolated from *A. compacta* and not from *A. atacamensis*. Assuming that mulinane-type diterpenoids are responsible for the observed biological activity, we assessed the anti-pyocyanin activity of all *A. atacamensis* fractions. The results reveal that the fractions enriched in mulinane-like diterpenoids and other terpene-type compounds are active and exhibited effective anti-pyocyanin activity (>50% of inhibition), while fractions less enriched in terpene-type compounds are less or non-active towards the inhibition of pyocyanin production. A similar tendency of results was observed when representative *A. atacamensis* enriched and less-enriched fractions in mulinane-like diterpenoids were assessed for elastase and rhamnolipid production. Overall, *A. atacamensis* appears to be an important source of mulinane-type diterpenoids. Our findings suggest that the anti-virulence properties might possibly be attributed to this singular interesting class of phytochemicals. Therefore, a synergistic effect can be assumed between all the mulinane-like diterpenoids present in the extract or fractions, as being responsible for the anti-virulence activity against *P. aeruginosa*. To confirm this hypothesis, further research should be undertaken to isolate and identify these interesting bioactive compounds and to confirm their effect alone or in combination as cocktails.

Terpenoids and derivatives such as sesquiterpenoids, diterpenoids, and triterpenoids have been reported to target pigments, exoenzymes, and surfactants production by *P. aeruginosa*. Among terpenoid class compounds, farnesol, a sesquiterpene synthesized by a variety of organisms, and other isoprenoid derivatives, including farnesyl acetate and geranyllinalool, were shown to be able to attenuate *P. aeruginosa* PA14 pyocyanin production [67]. Parthenolide, a sesquiterpene lactone possessing a gamma lactone moiety derived from *Tanacetum parthenium*, was also shown to attenuate pyocyanin pigment and protease production [68]. Sesquiterpene lactones isolated from *Centratherum punctatum* ssp. *punctatum* attenuated elastase production in *P. aeruginosa* ATCC 27,853 [69]. Dehydroleucodine, a sesquiterpene lactone isolated from *Artemesia douglasiana*, attenuated significantly LasA staphylolytic and LasB elastase activities [70]. The diterpene phytol possesses a pyocyanin inhibitory effect [71]. Moreover, phytol isolated from ethanolic extract from the leaves of *Syzygium jambos* and *Syzygium antisepticum* plants has been identified as an inhibitor of protease activity in a dose-dependent manner [72]. Furthermore, diterpenoids such as 14-deoxy-11,12-didehydroandrographolide and andrographolide isolated from *Andrographis paniculata*, also known as the “King of Bitters”, were shown to attenuate pyocyanin production and LasB elastase and exoprotease activities of *P. aeruginosa* MTCC 7814 without showing any antimicrobial activity. The inhibition of elastase and protease production was more significant in the presence of 14-deoxy-11,12-didehydroandrographolide as compared to cultures of *P. aeruginosa* MTCC 7814 treated with andrographolide. Interestingly, both compounds exhibited synergistic effects when combined with azithromycin or gentamicin [73]. Rajkumari and colleagues showed that betulin and betulinic acid found in many plants of the *Betulaceae* family decrease pyocyanin production without any effect on cell viability. These pentacyclic triterpenes reduced staphylolytic protease, LasA protease, LasB elastase and chitinase activities. Moreover, the authors reported that these triterpenoids decrease mortality rate in *P. aeruginosa*-infected *C. elegans* worms [74]. Various triterpenes and aromadendrane sesquiterpenes isolated from an Argentinian Liverwort *Lepidozia chordulifera* were found to reduce elastase production by *P. aeruginosa* ATCC 27853. Among them, ursolic and betulinic acids showed the strongest inhibition activity of elastase production [75]. In addition, a study conducted by Rasamiravaka and colleagues showed that cassipourol and β-sitosterol, two terpenoids from *Platostoma rotundifolium* (Briq.) A. J. Paton, reduced the production of rhamnolipids [76]. Herein, we report the anti-virulence effect of a singular class of diterpenoids which rely on the mulinane-like skeleton, and they deserve further work to establish their mode of action.

To understand the mechanism of action underlying the anti-virulence properties of *Aa*E and its active fractions, we attempted to investigate their effect on *P. aeruginosa* cell envelope using fluorescence anisotropy. The cell envelope represents the first contact surface between bacteria and their surrounding microenvironment, and thus is a highly selective barrier. Moreover, the cell envelope is known to be involved in maintaining cell structure and morphology, acquisition of nutrients and protection from toxic substances such as antibiotics. The interaction between plant-derived phytochemicals and biological membranes can lead to modifications of the membranes properties, directly via their insertion into the lipid bilayer membranes, or indirectly by altering membranes homeostasis leading to a bacterial response which in turn would disturb membrane’s fluidity. Terpenoids were shown to modify membrane properties and organization and have been reported to be involved in their antifungal, antibacterial, antiparasitic, and antioxidant, among other activities [77]. The *Aa*E and its most active fractions *Aa*F-2 and *Aa*F-6 (enriched-diterpene fractions) induced membrane rigidification of *P. aeruginosa* but not the non-active fraction *Aa*F-12. These data reveal that mulinanes-type compounds present in *Aa*E and its fractions *Aa*F-2 and *Aa*F-6 might interact with *P. aeruginosa* membranes lipid bilayers, hence triggering membrane homeostasis alteration. Therefore, this phenomenon seems to be modulated by the ECFσ SigX cell wall stress response, since *Aa*E and its enriched fractions on mulinane-type diterpenoids had no effect on membrane homeostasis of a Δ*sigX* deletion mutant strain. The ECFσ SigX is known to play a major role in the control of the composition membrane lipid and in the maintenance of *P. aeruginosa* cell wall homeostasis as well as its virulence traits [57,78,79,80,81]. While these data suggest that mulinane-like diterpenoids interfere with *P. aeruginosa* virulence through a potential mechanism partly involving the ECFσ SigX, further investigations will be required to elucidate the specific molecular mechanism of action.

## 5. Conclusions

Altogether, according to the literature, this study reports for the first time the anti-virulence activity of the medicinal plant *Azorella atacamensis* against the human opportunistic pathogen *Pseudomonas aeruginosa*. Interestingly, the mulinane-like diterpenoids putatively identified from *A. atacamensis* appear to be responsible for the observed virulence attenuation. However, further research will focus on the purification and structure identification of these singular mulinane-like bioactive compounds and determine their effect alone or in synergy as well as their antibiotic-potentiating effect and intrinsic bacterial resistance interference in *P. aeruginosa* multidrug resistant clinical isolates. Another important finding of the present study was that membrane homeostasis in *P. aeruginosa* has been significantly altered upon exposure to *A. atacamensis* extract. These findings suggest that mulinane-like diterpenoids may act as bioactive membrane-interactive compounds, which might trigger a cell wall stress response through the modulation of the ECFσ SigX. Therefore, the elucidation of molecular mechanisms of mulinanes-like diterpenoids through omics approaches may eventually pave the way for the identification of novel bacterial targets.

## Figures and Tables

**Table 1:** Dereplicated compounds in the ethyl acetate extract and fractions of *Azorella atacamensis*.

ID	[M-H]^−^ *m*/*z*	Δppm	Molecular Formula	R_T_ (min)	Putative Identity	DNP-ID ^a^	Biological Source ^b^	Identification Level [56]
1	335.2230	0.4	C_20_H_32_O_4_	2.79	13,14-Dihydroxy-11-mulinen-20-oic acid	NYP46	*Mulinum spinosum Azorella compacta*	2
2	333.2075	2.3	C_20_H_30_O_4_	3.27	Isomulinic acid	KKF97	*Azorella atacamensis*	2
3	333.2074	0.6	C_20_H_30_O_4_	3.34	13-Hydroxy-14-oxo-11-mulinen-20-oic acid	QKS62	*Azorella madreporica*	2
4	333.2074	0.6	C_20_H_30_O_4_	3.44	11,14-Dioxo-12-mulinen-20-oic acid; 11a-Alcohol	RRT66	*Azorella trifurcata*	2
5	331.1920	1.2	C_20_H_28_O_4_	3.75	11,14-Dioxo-12-mulinen-20-oic acid	HFY52	*Azorella compacta*	2
6	319.2283	1.2	C_20_H_32_O_3_	3.83	13-Hydroxy-11-mulinen-20-oic acid	JON06	*Azorella atacamensis*	2
7	391.2129	1.4	C_22_H_32_O_6_	3.91	17-Acetoxymulinic acid	PCM78	*Azorella atacamensis*	2
8	433.2233	2.8	C_24_H_34_O_7_	3.92	Mulinone B	RLQ24-N	*Azorella atacamensis*	2
9	333.2076	1.7	C_20_H_30_O_4_	4.18	Mulinic acid	KKF96	*Azorella atacamensis*	1
10	317.2125	1.3	C_20_H_30_O_3_	4.28	11,12-Epoxy-13-mulinen-20-oic acid	FKO04	*Azorella compacta*	2
11	315.1971	1.5	C_20_H_28_O_3_	4.42	11-Oxo-12,14-mulinadien-20-oic acid	HFY53	*Azorella compacta*	2
12	317.2125	1.2	C_20_H_30_O_3_	4.75	13,17-Epoxy-11-mulinen-20-oic acid (Mulinenic acid)	KTX88	*Azorella atacamensis*	2
13	359.2232	1.5	C_22_H_32_O_4_	5.29	17-Acetoxy-11,13-mulinadien-20-oic-acid	FRY02-H	*Azorella atacamensis*	2
14	301.2177	1.3	C_20_H_30_O_2_	5.77	11,13-Mulinadien-20-oic acid	PCR06	*Mulinum spinosum Azorella compacta*	2

^a^ CRC code in the Dictionary of Natural Products v29.1. ^b^ Plant species reported as a source of the compound in the Dictionary of Natural Products v29.1.

## Supplementary Materials

The following are available online at https://www.mdpi.com/2218-273X/10/12/1626/s1, Figure S1: Chromatographic traces: Total ion current chromatogram ESI positive (orange), Total ion current chromatogram ESI negative (blue) and Charged Aerosol Detector (CAD, lower), Figure S2: Molecular network in negative ionization mode for the *Azorella atacamensis* extract (*Aa*E), Figure S3: The fraction *Aa*F-4 analyzed by a preparative HPLC on an AP-MOD-100 apparatus (Armen Instrument, Saint-Avé, France) with a c18 PursuitTM Varian column (250 mm × 30 mm, 10 µm), Figure S4: Combined molecular network in negative ionization mode for the ethyl acetate extract and fractions of *Azorella atacamensis*, Table S1: Reported compounds in the Dictionary of Natural Products (DNP v29.1) for the genus *Azorella* and *Mulinum*.
