# Supplementary material for: Tackling Pseudomonas aeruginosa Virulence by Mulinane-Like Diterpenoids from Azorella atacamensis
Source: Biomolecules. 2020 Dec 2;10(12):1626. doi: 10.3390/biom10121626 (PMC7761567; doi:10.3390/biom10121626)
Supplement: Supplementary file 1 [file biomolecules-10-01626-s001.pdf]

biomolecules

MDPI

Supplementary Materials

Tackling *Pseudomonas aeruginosa* Virulence by Mulinane-like Diterpenoids from *Azorella atacamensis*

Onyedikachi Cecil Azuama [1,2], Sergio Ortiz [3], Luis Quirós-Guerrero [4,5], Emeline Bouffartigues [1], Damien Tortuel [1], Olivier Maillot [1], Marc Feuilloley [1], Pierre Cornelis [1], Olivier Lesouhaitier [1], Raphaël Grougnet [2], Sabrina Boutefnouchet [2], Jean-Luc Wolfender [4,5], Sylvie Chevalier [1] and Ali Tahrioui [1,*]

*corresponding author:
Dr. Ali Tahrioui
Laboratory of Microbiology Signals and Microenvironment–LMSM EA4312,
University of Rouen Normandy–Normandy University,
55 Rue Saint-Germain, 27000 Evreux, France
E-mail: ali.tahrioui@univ-rouen.fr
Phone: (+33) 2.32.29.15.60 - Fax: (+33) 2.32.29.15.50

biomolecules

MDPI

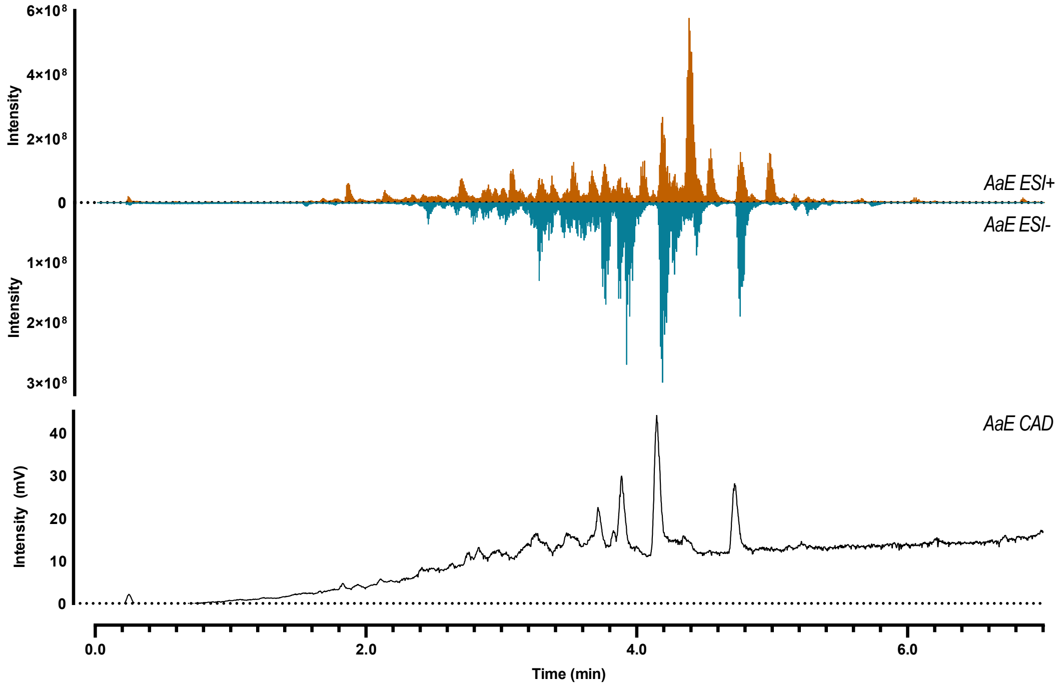

Figure S1. Chromatographic traces: Total ion current chromatogram ESI positive (orange), Total ion current chromatogram ESI negative (blue) and Charged Aerosol Detector (CAD, lower).

biomolecules

MDPI

Table S1. Reported compounds in the Dictionary of Natural Products (DNP v29.1) for the genus *Azorella* and *Mulinum*. In bold, the compounds previously reported in the species studied in this work.

Chemical Name	Molecular Formula	Accurate Mass	Biological source
4,5,11-Guaianetriol; (1a,4b,5b,10b)-form	$C_{15}H_{28}O_3$	256.203845	*A. cryptantha*
2,9-Pentadecadiene-4,6-diyne-1,8-diol; (Z,Z)-form, 1-Ac	$C_{17}H_{22}O_3$	274.156895	*A. trifurcata*
2,9-Heptadecadiene-4,6-diyne-1,8-diol; (2Z,8S,9Z)-form	$C_{17}H_{24}O_2$	260.17763	*A. trifurcata*
2,9-Heptadecadiene-4,6-diyne-1,8-diol; (2Z,8S,9Z)-form, 1-Ac	$C_{19}H_{26}O_3$	302.188195	*A. trifurcata*
Madreporanone	$C_{19}H_{32}O_3$	308.235145	*A. madreporica*
Yaretol	$C_{19}H_{32}O_3$	308.235145	*A. madreporica*
11-Oxo-12,14-mulinadien-20-oic acid	$C_{20}H_{28}O_3$	316.203845	*A. compacta*
11,14-Dioxo-12-mulinen-20-oic acid	$C_{20}H_{28}O_4$	332.19876	*A. compacta*
11,13-Mulinadien-20-oic acid	$C_{20}H_{30}O_2$	302.22458	*M. spinosum, A. compacta*
Azorellolide	$C_{20}H_{30}O_2$	302.22458	*A. cryptantha*
11,12-Epoxy-13-mulinen-20-oic acid; (11a,12a)-form	$C_{20}H_{30}O_3$	318.219495	*A. compacta*
11,13-Mulinadien-20-oic acid; 17-Hydroxy	$C_{20}H_{30}O_3$	318.219495	*M. spinosum*
14-Hydroxy-11,13(16)-mulinadien-20-oic acid; 14a-form	$C_{20}H_{30}O_3$	318.219495	*A. trifurcata*
14-Oxo-12-mulinen-20-oic acid	$C_{20}H_{30}O_3$	318.219495	*M. spinosum*
16-Oxo-12-mulien-20-oic acid	$C_{20}H_{30}O_3$	318.219495	*A. madreporica*
**Mulinenic acid**	**$C_{20}H_{30}O_3$**	**318.219495**	***M. crassifolium***
11,14-Dioxo-12-mulinen-20-oic acid; 11a-Alcohol	$C_{20}H_{30}O_4$	334.21441	*A. trifurcata*
13-Hydroxy-14-oxo-11-mulinen-20-oic acid; 13a-form	$C_{20}H_{30}O_4$	334.21441	*A. madreporica*
**Isomulinic acid**	**$C_{20}H_{30}O_4$**	**334.21441**	***M. crassifolium***
**Mulinic acid**	**$C_{20}H_{30}O_4$**	**334.21441**	***M. crassifolium***
13,17-Azorellanediol; 13b-form, 17-Aldehyde	$C_{20}H_{32}O_2$	304.24023	*A. cryptantha*
7,13-Azorellanediol; (7b,13a)-form, 7-Ketone	$C_{20}H_{32}O_2$	304.24023	*A. yareta*
Azorellolide; 17-Alcohol (lactol)	$C_{20}H_{32}O_2$	304.24023	*A. cryptantha*
**13-Hydroxy-11-mulinen-20-oic acid; 13a-form**	**$C_{20}H_{32}O_3$**	**320.235145**	***M. crassifolium***
13,14-Dihydroxy-11-mulinen-20-oic acid; (13a,14a)-form	$C_{20}H_{32}O_4$	336.23006	*M. spinosum, A. compacta*
9,12-Cyclomulin-13-ol; 13b-form	$C_{20}H_{34}O$	290.260965	*A. madreporica*
11-Mulinene-13,20-diol	$C_{20}H_{34}O_2$	306.25588	*A. compacta*
**11,13-Mulinadien-20-oic acid; 17-Acetoxy**	**$C_{22}H_{32}O_4$**	**360.23006**	***M. crassifolium***
15-Hydroxy-11,13-mulinadien-20-oic acid; 15a-form, Ac	$C_{22}H_{32}O_4$	360.23006	*A. trifurcata*
**11,14-Epidioxy-17-hydroxy-12-mulinen-20-oic acid; Ac**	**$C_{22}H_{32}O_6$**	**392.21989**	***M. crassifolium***
11,13-Mulinadien-20-oic acid; 20-Alcohol, 20-Ac	$C_{22}H_{34}O_2$	330.25588	*A. compacta*
9,12-Mulinadien-7-ol; 7b-form, Ac	$C_{22}H_{34}O_2$	330.25588	*A. compacta*
11-Mulinene-7,13-diol; (7b,9a,13b)-form, 7-Ac	$C_{22}H_{36}O_3$	348.266445	*A. trifurcata*
13,17-Azorellanediol; 13a-form, 17-Ac	$C_{22}H_{36}O_3$	348.266445	*A. madreporica*
7,13-Azorellanediol; (7b,13a)-form, 7-Ac	$C_{22}H_{36}O_3$	348.266445	*A. compacta*
**14,17-Dihydroxy-11-oxo-12-mulinen-20-oic acid; 14a-form, 11a-Alcohol, 14-ketone, di-Ac**	**$C_{24}H_{34}O_7$**	**434.230455**	***M. crassifolium***
**14,17-Dihydroxy-11-oxo-12-mulinen-20-oic acid; 14a-form, Di-Ac**	**$C_{24}H_{34}O_7$**	**434.230455**	***M. crassifolium***

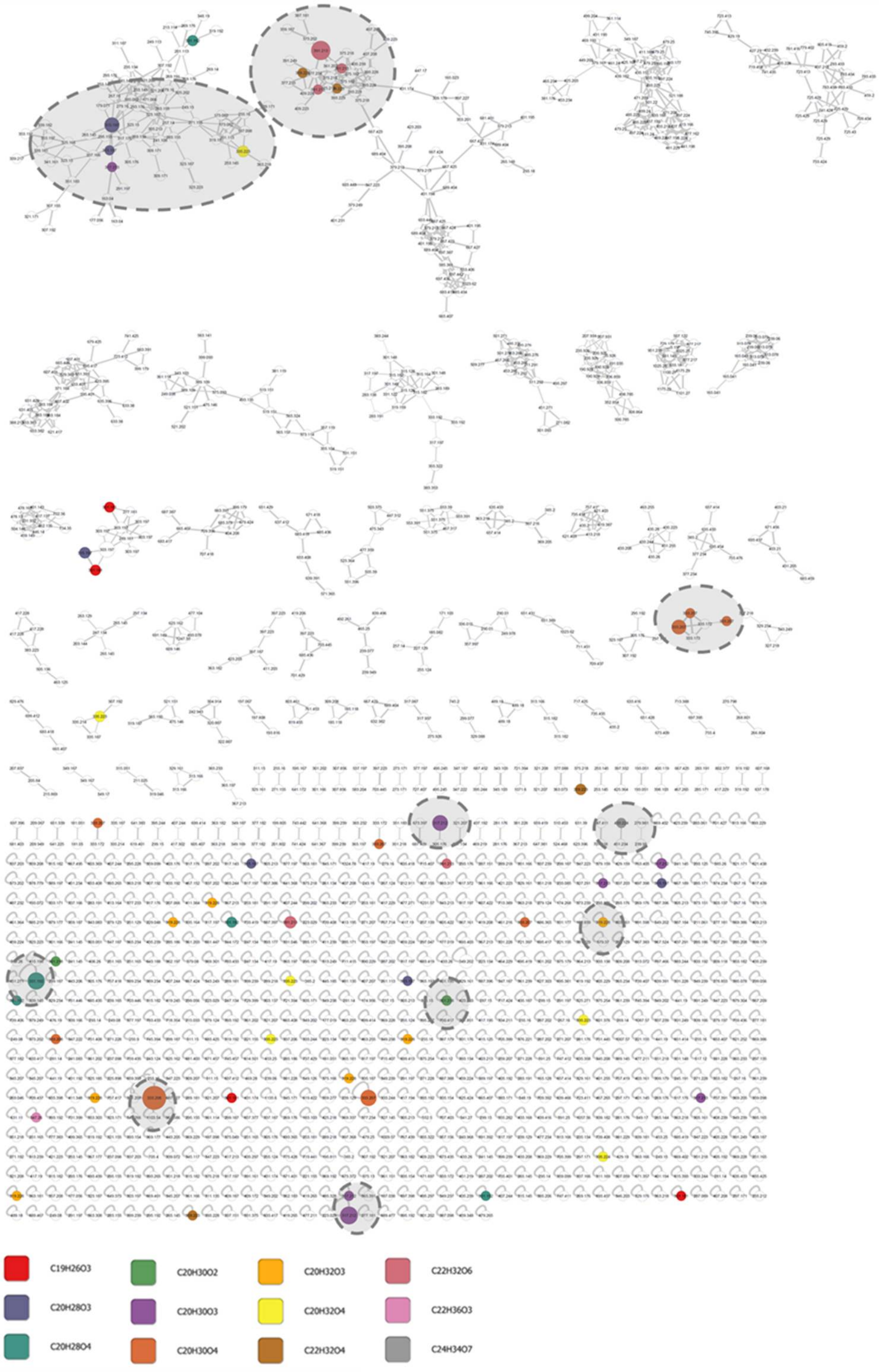

Figure S2. Molecular network in negative ionization mode for the *Azorella atacamensis* extract (*Aa*E). The displayed structures correspond to the fourteen putative identified compounds (1-14, see Table 1). The identification of mulinic acid (9) was confirmed by isolation (see Supplementary Figure S3). Numbers inside de nodes correspond to the precursor mass for each feature, and size is proportional to the intensity of each ion in the total ion current chromatogram of the extract. Color code use to represent the different general molecular formulas for all the reported compounds in the genus *Azorella* and *Mulinum* is shown below. The 'highlighted' clusters correspond to those magnified in the Figure 5 in the main text.

(**a**)

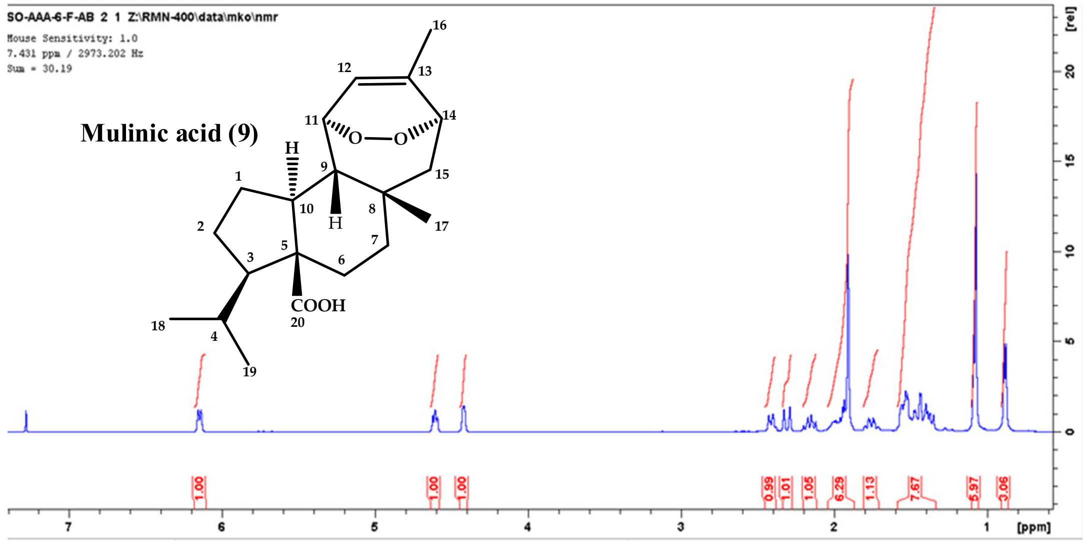

(**b**)

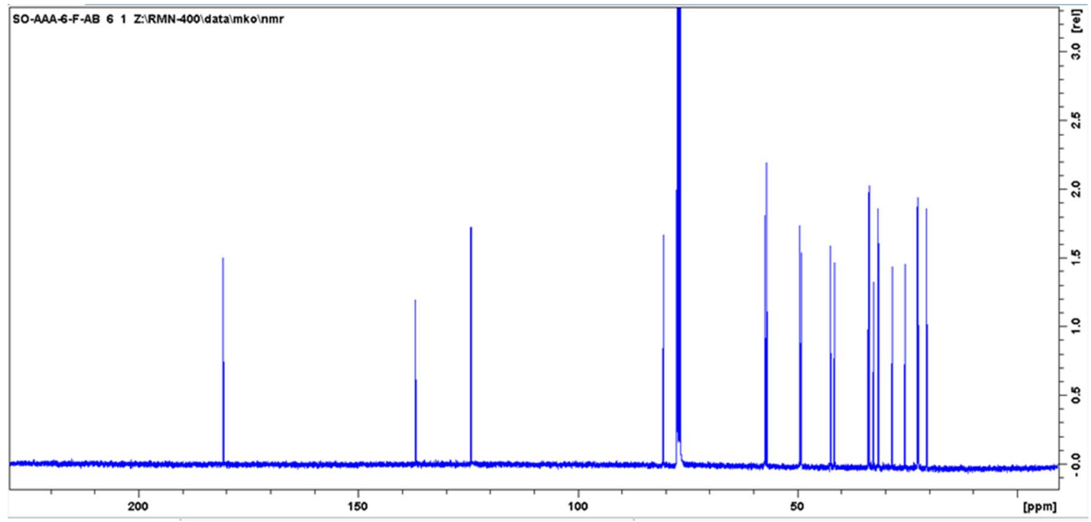

Figure S3. The fraction *Aa*F-4 analysed by a preparative HPLC on an AP-MOD-100 apparatus (Armen Instrument, Saint-Avé, France) with a c18 PursuitTM Varian column (250 mm x 30 mm, 10 μm). Isocratic conditions were applied (H2O/MeOH 8/2) and 15 mg of fraction *Aa*F-4 were submitted to purification. Compound (9) (8.5 mg) was isolated as white powder and identified as mulinic acid after comparison of its experimental and literature NMR data. (**a**) ^{1}H-NMR spectrum of mulinic acid (9) in $CDCl_3$ (400 MHz). δ 6.49 [d, *J* = 7.1 Hz, 1H, H-12], 4.59 [t, *J* = 6.6 Hz, 1H, H-11], 4.42 [d, *J* = 4.2 Hz, 1H, H-14], 2.43-2.38 [m, 1H, H-H-7α], 2.29 [d, *J* = 15.8 Hz, 1H, H-15α], 2.20-2.12 [m, 1H, H-10], 2.05-1.88 [m, 6H, 3H-16, H-1α, H-2α, H-9], 1.81-1.71 [m, 1H, H-1β], 1.53-1.35 [m, 7H, H-2β, H-3, H-4, H-6α, H-6β, H-7β, H-15β], 1.07 [s, 6H, 3H-17, 3H-18], 0.88 [d, *J* = 6.6 Hz, 3H-19]. (**b**) ^{13}C-NMR spectrum of mulinic acid in (9) $CDCl_3$ (125 MHz). δ (ppm) 180.8 (C-20), 136.9 (C-13), 124.3 (C-12), 80.5 (C-14), 77.6 (C-11), 57.5 (C-3), 57.0 (C-5), 49.5 (C-9), 49.1 (C-10), 42.5 (C-15), 41.6 (C-6), 33.8 (C-17), 33.6 (C-8), 32.7 (C-7), 31.6 (C-4), 28.4 (C-2), 25.5 (C-1), 22.7 (C-18), 22.5 (C-19), 20.6 (C-16).

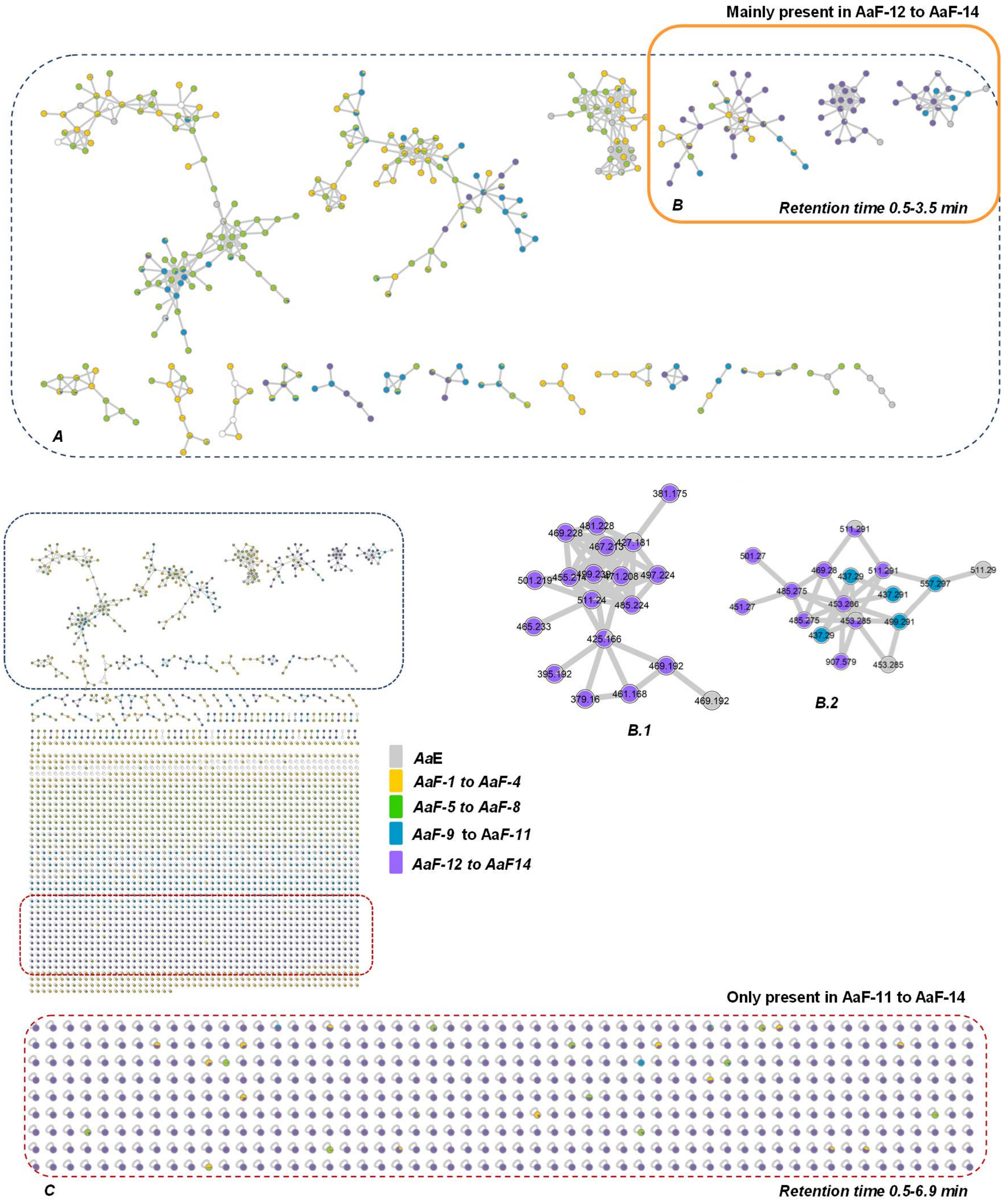

Figure S4. Combined molecular network in negative ionization mode for the ethyl acetate extract and fractions of *Azorella atacamensis*. Numbers inside the nodes correspond to the precursor mass for each feature. Colors represent the different fractions according to the code shown. **A**. Close-up of the upper section of the network. **B**. Selection of two specific clusters to the group *Aa*F-12 to *Aa*F-14. **B.1** and **B.2** Close-up of the clusters highlighted in section **B**. specific cluster to fractions *Aa*F-12 to *Aa*F-14. **C**. Close-up of the singletons in the middle section of the network specific to fraction *Aa*F-12 to *Aa*F-14.
